# Social mobility and perinatal depression in Black women

**DOI:** 10.3389/frhs.2023.1227874

**Published:** 2023-08-24

**Authors:** Melissa Hawkins, Arun Mallapareddi, Dawn Misra

**Affiliations:** ^1^ Department of Health Studies, College of Arts and Sciences, American University, Washington, DC, United States; ^2^ Department of Family Medicine and Public Health Sciences, School of Medicine, Wayne State University, Detroit, MI, United States

**Keywords:** social mobility, economic mobility, mental health, pregnancy, black, depression, CES-D

## Abstract

**Background:**

Higher socioeconomic position is associated with better birth outcomes and maternal mental health, although this relationship is less consistent for Black women. The literature is limited on the impact of social mobility across the life course on mental health of pregnant women. This study examines the impact of perceived financial status across the life-course on depressive symptoms during pregnancy among Black women.

**Methods:**

Data were from the Life-course Influences of Fetal Environments (LIFE) retrospective cohort study among pregnant Black women in metropolitan Detroit, Michigan. Depressive symptoms in the two weeks prior to birth were assessed using the Center for Epidemiologic Studies Depression (CES-D) scale. Social mobility was determined at three intervals over the life course using self-report of financial status during childhood, adolescence, and current age in pregnancy.

**Results:**

1,410 pregnant women participated, ranging in age from 18 to 45 years old. CES-D scores ranged from 0 to 53 (mean = 15.3) and 26% of the sample reported high depressive symptoms. In each age interval, higher financial status was associated with significant protective effect on depressive symptoms, and the magnitude of the effect increased across the life course. Trajectory analysis demonstrated that both the upward (4.51; 95% CI, 2.43–6.6) and downward (4.04; 95% CI, 2.62–5.46 and 3.09; 95% CI, 1.57–4.62) life-course social mobility groups had increased mean CES-D scores compared to the static social mobility group.

**Conclusion:**

This study describes the importance of previous childhood and current financial status effects on mental health in Black pregnant women.

## 1. Introduction

Pregnancy is a time of significant change in a woman's life course. The prevalence of depression in pregnancy is estimated to be 10%–15% (1), and Black women experience higher levels of depressive symptoms in pregnancy compared to white women (2–4). Depression during pregnancy is associated with adverse birth outcomes, including preterm birth (<37 weeks completed gestation) and low birth weight (<2,500 grams) (5–7). Rates of depression are increasing in the United States (US), particularly among women and racial/ethnic minority groups (8). Depressive symptoms vary in severity and are characterized by loneliness, loss of interest in activities, and somatic dysfunctions (e.g., loss of appetite and sleep disturbances) (9).

There is a consistent and direct association between socioeconomic position (SEP), typically measured by education, income, or occupational class, and health; high SEP is associated with better health (10). There is an inverse relationship between depression prevalence and SEP, with lower SEP associated higher rates of depression (11). Pregnant women with low SEP report significantly more depressive symptoms (12) than women with higher SEP. Although maternal low SEP and previous chronic conditions may increase the risk of depressive symptoms, these factors do not fully account for the disparities in depressive symptoms (13, 14). There is growing evidence that the disproportionate poor pregnancy outcomes among Black women are attributable to social and psychosocial factors rather than biologic differences that mediate inequities (15–17). Further, although the association between SEP and health has been consistently shown to have a positive linear relationship (18), a growing body of literature indicates that benefits of upward SEP on health may be less substantial for Black women compared with white women (19–21) through limited access to resources and opportunities (22, 23).

Few studies have evaluated SEP over the life course and the association with depressive symptoms (24–27), and the results are inconsistent for Black women (21). Recently, Miller et al. (28) found that both downward and upward socioeconomic mobility was associated with depressive symptoms. Slaughter et al. (29) found a protective effect on birth outcomes among Black women who experienced upward mobility in their SEP between childhood and adulthood, suggesting the effects may have been driven, in part, by depressive symptoms.

Elucidating the mechanisms that contribute to persistent health disparities in the US is further complicated when considering financial status and sex across the life course (30). That is, SEP operates differently between men and women (31) with research consistently demonstrating that women report more stressful events and adversity in childhood compared to males (32, 33). These findings highlight the importance of childhood financial circumstances on later adult health and also acknowledge gender disparities which has implications for reproductive justice efforts (34, 35).

Compared to other developed countries, the US has greater income inequality and rates of upward economic mobility are relatively low (36). However, the literature is limited on how changes in SEP or financial status over the life course impacts mental health in adulthood or in pregnancy. Social mobility is defined as the movement of social status of a person between different strata over time. Mobility can be upward or downward (37). Nicklett and Burgard (38) found that a downward intra-generational social mobility increases the risk of developing depression in adulthood. Economic mobility is a component of social mobility specific to movement between economic strata (39). Economic mobility can encompass the changes in a person's financial status over their life-course. Life course financial status has been previously shown to have an effect on health in adulthood, with lower life course SES having a deleterious effect (40–42).

The literature examining the association between SEP, social mobility and depressive symptoms during pregnancy is inconsistent with mixed findings (18), however. Relatively few studies have examined life course SEP mobility and depressive symptoms among Black women in the US (18). Recently, Hoven (43) described patterns of social mobility and their associations with depressive symptoms through a life course perspective from early and mid-adulthood to later ages (45–60 years) among a large French cohort and did not find a consistent association between social mobility and health, either upward or downward mobility. However, studies incorporating life course SEP and social mobility may provide important information on etiology that are relevant for prevention efforts, and in explaining disparities in birth outcomes (44). Mental health and maternal depression, in particular, may be a limiting factor in economic mobility.

Measurement of SEP and social mobility is challenging. SEP is a broad concept and includes both financial and non-financial constructs. The operationalization of SEP has traditionally represented individual level economic status using data on occupation, education level and/or income. Recent research has demonstrated that the association between mental health and financial status is stronger than the association between mental health and either education or social class (45). Therefore, an increased understanding of the impact of financial status in childhood and adolescence on adult health is necessary. Osypuk et al. (46) operationalized social mobility as subjective self-report of financial status. Chetty et al. (47) argue that the most robust approach to measure intergenerational mobility is a rank-rank method where the individual ranks parents by parental income during childhood and to rank their income as adults. Prior studies often use proxies to calculate childhood SEP (48) which may introduce measurement error.

Three primary frameworks have emerged in life course epidemiology that elucidate how duration, changes, and timing of SEP impacts health across time (49, 50): (1) critical period framework posits that SEP in early life influences health later via sensitive developmental periods; (2) risk accumulation framework suggests that the impact of SEP is cumulative over time to impact health; and (3) more recently, social mobility framework proposes that upward or downward change in SEP between life stages influences health (51). That is, improved social mobility may improve health outcomes by decreasing risk or prolonged exposure to adverse exposures over the life course, and upward mobility may compensate for early adverse life exposures. In contrast to the critical period framework, the social mobility model predicts that early-life effects are modified by later SEP and emphasizes that transition between advantage and disadvantage (52). The social mobility framework guides this work, based on the measurement of perceived financial status at three time intervals over the life course among a cohort of pregnant Black women.

This study examines the impact of economic mobility (using perceived measures of financial status) of Black pregnant women during childhood, adolescence and currently in adulthood on depressive symptoms during pregnancy. The study seeks to elucidate the economic factors associated with depression during pregnancy. Social mobility is characterized by three different intervals in the life course, which provides a more comprehensive account of SEP beyond a single time period at birth, childhood, or adulthood alone. The primary objectives of this study were to 1) describe associations between individual SEP indicators and depressive symptoms of Black women in pregnancy and 2) assess the association between social mobility from childhood to adolescence and adulthood with depressive symptoms.

## 2. Materials and methods

This study is a secondary analysis of the Life-course Influences on Fetal Environments (LIFE) study which examined social and biomedical factors that influence preterm birth among Black women. The methodological approach of the LIFE study have been previously described (53, 54). In brief, the LIFE study is a retrospective cohort of 1,410 pregnant Black women recruited from June 2009–December 2011 from the postpartum unit of a suburban hospital in Metropolitan Detroit, Michigan. Eligibility criteria for participation included: self-identified as Black or African American, 18–45 years old, English speaking, and had a singleton birth. Exclusion criteria included: intellectual disability, serious cognitive deficits, or diagnosis of significant mental illness, based on previous medical history.

Data were collected between 24 and 72 h after birth. Women participated in structured in-person interviews with trained interviewers in their hospital room (see Supplementary Appendix S1 for full interview transcript). Trained interviewers were matched to study participants on race and gender to conduct interviews (45–60 min long) on sociodemographic, psychosocial, and biomedical factors over the life course. Obstetrical and medical history was abstracted from the medical records by the research staff. A $50 gift card was provided as an incentive for study participation.

The study protocol was approved by the university and hospital Institutional Review Board. All study participants provided written informed consent prior to data collection. The final sample includes 1,410 women, which represented 71% of the women invited to participate.

### 2.1. Measures

#### 2.1.1. Depression

Depressive symptoms were assessed using the validated Center for Epidemiologic Studies Depression Scale (CES-D) (55, 56) for use in the general population. The 20-item CES-D screens for depressive symptoms in the previous 7 days (e.g., *I felt depressed*, *I had crying spells*). Each item response is on a 4-point Likert scale (0 = *rarely* or none of the time to 3 = *most or all of the time*) with scores ranging from 0 to 60. A higher score indicates the presence of more depressive symptoms. In this analysis, the dichotomized score of 23 or higher was used to indicate clinically significant prenatal depressive symptoms (57) given the symptoms of pregnancy, such as fatigue or change in sleep or appetite, overlap with the symptoms of depression. The measure is highly reliable as reflected in the current study's Cronbach's alpha of 0.89.

#### 2.1.2. Social mobility

Three questions from the National Survey of American Life were used to assess the self-reported perceived finances during two childhood intervals (early childhood from birth to age 10 years) and adolescence (from age 10 to 18 years) and currently in adulthood (58) including: (1) How would you describe your family's financial situation today?; (2) How would you describe your family's financial situation between the time you were born (birth) and your 10th birthday?, and; (3) How would you describe your family's financial situation between your 10th and 18th birthdays? Item responses were on a 5-point Likert scale (1 = *very poor, not enough to get by*; 2 = *barely enough to get by*; 3 = *enough to get by but no extras*; 4 = *more than enough to get by*; and 5 = *well-to-do)*. Higher values indicate better financial status (59). An increase in the financial status from an age interval to older age intervals was defined as upward economic mobility and a decrease as a decline in economic mobility. Mobility scales were created to assess change in perceived financial status from adulthood current to the two childhood intervals. Social mobility was calculated as the difference of adulthood minus childhood and adolescent financial status. Women's current situation in adulthood compared with previous intervals, with negative values indicating that the woman exhibited downward social mobility and positive values indicating upward social mobility. Zero indicated static financial status. Financial status differences between age intervals were calculated.

### 2.2. Statistical analysis

All interview data were entered, cleaned, and checked for accuracy by trained research assistants. Descriptive analysis was conducted to summarize maternal sociodemographic characteristics, depressive symptoms (CES-D) and financial status across the life course. The mean and standard deviation (SD) for each of the measures and social mobility scales (both total score and each item response) was calculated. Financial status groups were combined to create three groups (poor, enough to get by, and well to do) for some analyses due to distribution of the data. All statistical analyses were performed using IBM SPSS Statistics for Windows, Version 25.0 (Armonk, NY: IBM Corp) and SAS software, Version 9.4 (SAS Institute, Cary, NC, USA).

Multiple linear regression was performed to examine the relationship between the financial status at the three life course intervals and the aggregate depressive symptom scores, as well as social mobility change and depressive symptoms scores. Maternal age was examined as a potential confounder in regression analyses. Group-based trajectory modeling is gaining popularity in epidemiology. Group-based trajectory modeling was conducted to examine the changes in financial status over the three times over the life course with other covariates. The social mobility groups from the trajectory analysis were used as predictors in the multiple linear to regression with CES-D scores as the outcomes. The trajectory analysis assumes that the population is composed of distinct socioeconomic groups, each with a different underlying trajectory. The aim of this analysis is to identify groups following similar SEP over time and then estimate the effects of covariates on the trajectory and group characteristics. As such, it is assumed that time dependent covariates explain variation about the average trajectory within each group or their equivalents for your field.

## 3. Results

### 3.1. Sociodemographic characteristics

A total of 1,410 women participated in this study. Sociodemographic characteristics and prevalence of adverse birth outcomes among LIFE study participants were comparable to Black women in who gave birth in the US, the state of Michigan, and Detroit metropolitan area in 2010 (29, 46). Sixteen percent (*n *= 230) of the participants had a preterm birth and 43% (*n *= 602) of the participants had first birth. The study participants had a mean age of 27.3 ± 6.2 years. The majority of women had a high school degree or higher education (*n *= 845; 71.7%) and were married or cohabiting (*n *= 621; 53.1%) (Table 1).

**Table 1:** Demographic characteristics of study participants (*n *= 1,410).

Characteristic	No.	Valid %
Age		
18–19	119	8.5
20–24	433	30.3
25–29	373	26.5
30–34	270	19.1
≥35	215	15.3
Marital status^a^		
Single	655	46.8
Married or cohabitating	744	53.2
Education, years		
≤12	398	28.2
>12	1,012	71.8
Residence^a^		
City of Detroit	666	49.0
Detroit suburb	693	51.0

^a^ 11 women were missing data on marital status; 51 women were missing data on residence.

### 3.2. Perinatal depression

Depressive symptoms were prevalent among the participants. CES-D scores ranged from 0 to 53, with a mean score of 15.3. Forty-one percent of women (*n *= 580) had CES-D scores ≥16, which have been correlated with clinically relevant depression and 19.6% (*n *= 277) had scores >23, indicating high levels of depressive symptoms (60). Women with CES-D scores ≥16 were younger, were more likely to be single, and had lower levels of education and household income compared with women with CES-D <16.

### 3.3. Financial status and economic mobility

The majority of participants had an annual household income of $30,000 or more (*n *= 739; 53.4%). Table 2 details the SEP across the life course and Table 3 details the financial status items, including changes in economic mobility using both the 5-group scale for financial and the 3-group scale. Approximately 30% of the women reported that they “*had more than enough to get by*” in early childhood and adolescence time intervals. Approximately, 50% of women reported they “*had enough to get by*” currently in adulthood. Most of the participants did not experience change in their financial status before 18 years of age. Downward financial mobility is more prevalent during the later age intervals, from adolescence to adulthood (Table 4).

**Table 2:** Socioeconomic position (SEP) over the life course (*n *= 1,410).

Measure	Mean	SD	Range	% Missing
Financial status				
Current (adulthood)	3.42	0.81	1 to 5	0%
Birth to age 10 (early childhood)	3.65	0.96	1 to 5	1%
Age 10 to 18 (adolescence)	3.63	0.9	1 to 5	0%
Educational attainment				
Woman's own (adulthood)	2.84	0.88	1 to 4	0%
Woman's mother's (childhood SEP)	2.67	0.96	1 to 4	5%
Financial mobility				
Birth to current adulthood	−0.22	1.15	−4 to4	2%
Adolescence to current adulthood	−0.21	1.02	−4 to3	1%
Educational mobility	0.2	1.1	−3 to3	6%

**Table 3:** Financial Status over the life course (*n* = 1,410).

Financial Status (5-group scale)	Age Intervals		
	Early Childhood (Birth-10 years)	Adolescence (10–18 years)	Adulthood (current)
	*n* (%)	*n* (%)	*n* (%)
Poor (1)	32 (2.3%)	19 (1.3%)	20 (1.4%)
Barely enough (2)	129 (9.1%)	112 (7.9%)	92 (6.5%)
Had enough (3)	394 (27.9%)	472 (33.5%)	711 (50.4%)
Had more than enough (4)	584 (41.4%)	568 (40.3%)	445 (31.6%)
Well to do (5)	252 (17.9%)	232 (16.5%)	139 (9.9%)
Financial Status (3-group scale)	*n* (%)	*n* (%)	*n* (%)
Poor (1,2)	161 (11.4%)	131 (9.3%)	112 (7.9%)
Had enough (3)	394 (27.9%)	472 (33.5%)	711 (50.4%)
Well to do (4,5)	836 (59.2%)	800 (56.7%)	584 (41.4%)
Total	1,391 (98.7%)	1,403 (99.5%)	1,407 (99.8%)
Missing	19 (1.3%)	7 (0.5%)	3 (0.2%)

*n*(%), number of participants in each group (valid %).

**Table 4:** Economic mobility across life course.

Economic Mobility	EM from Early Childhood to Adolescence *n* (%)	EM from Adolescence to Current Adulthood *n* (%)
Downward	236 (16.7%)	436 (30.9%)
Static	916 (65.0%)	721 (51.1%)
Upward	235 (16.7%)	243 (17.2%)
*Total valid*	*1,387 (98.4%)*	*1,400 (99.3%)*
*Missing*	*23 (1.6%)*	*10 (0.7%)*
Total participants	1,410 (100%)	1,410 (100%)

*n*(%), number of participants in each group (valid %); EM, Economic Mobility using the 3-group scale for financial status.

### 3.4. Financial status and depression

Linear regression was used to examine the relationship between financial status (using the 3-group scale) over the life course with current depressive symptoms (CES-D scores) at each age interval (Table 5). Higher self-reported financial status was inversely related to depression and the protective effect increased over the life course. This effect is statistically significant during the adolescence age interval of 10 years to 18 years and current age interval (*p *< 0.05) but is not significant during early childhood birth to 10 years of age interval (*p *= 0.13). In the early childhood interval, a one-point increase in the financial status scale was associated with approximately a half point decrease in the CES-D (*p *< 0.05), while this same increase in the adolescent interval (10–18 years of age) was associated with approximately one point decrease in the CES-D (*p *< 0.05). The effect of current financial status in adulthood was the largest effect with a nearly two-point decrease in CES-D associated with a one-point increase in the financial score (*p *< 0.05).

**Table 5:** Linear regression: financial Status predicting CES-D score.

Life Course	*N*	Change in CESD for 1 point increase in Financial Status (95% CI)
Financial status with 5 groups (scored 1 to 5: very poor = 1; barely enough = 2; had enough = 3; had more = 4; well to do = 5)		
Early Childhood (Birth to 10)	1,391 (98.7%)	−0.42 (−0.96, 0.13)
Adolescence (10–18)	1,403 (99.5%)	−1.04 (−1.62, −0.46)^a^
Current Adulthood	1,407 (99.8%)	−1.87 (−2.50, −1.23)^a^
Financial status re-categorized into 3 groups (poor = 1; had enough = 2; well to do = 3)		
Early Childhood (Birth to 10)	1,391 (98.7%)	−0.86 (−1.61, −0.11)^a^
Adolescence (10–18)	1,403 (99.5%)	−1.53 (−2.32, −0.75)^a^
Current Adulthood	1,407 (99.8%)	−2.65 (−3.48, −1.82)^a^

^a^ *p *< 0.05. *n*-number of participants in each group; regression coefficient estimate of a change for a 1 point increase in financial status, using both 5-group and 3-group scales.

Table 6 shows the linear regression analysis for the economic mobility of each interval with depressive symptoms. It can be observed that in both of the childhood intervals, the downward economic mobility group has a significant negative effect on CES-D scores compared to the static financial group. The mean CES-D score for women in downward economic mobility group is 1.87 points higher than the static group within the early childhood and adolescence age interval, and 1.59 point higher during the adolescence to current adulthood interval. The upward economic mobility group in both the intervals also has a higher mean CES-D score, but these results were not statistically significant. When controlling for the effect of age, we see a protective effect of older age on CES-D scores, particularly for the upward social mobility group from middle in early childhood to high (group 2) (>10%).

**Table 6:** Linear regression: economic mobility predicting CES-D score.

Economic Mobility Group	*N* (%)	Differences in Mean CES-D (95 CI)
Early Childhood (birth-10 years) to Adolescence (10–18 years)		
Static	916 (65.0%)	REF
Downward	236 (16.7%)	1.863 (0.44, 3.29)^a^
Upward	235 (16.7%)	0.754 (−0.65, 2.16)
Adolescence (10–18 years) to Current Adulthood		
Static	721 (51.1%)	REF
Downward	436 (30.9%)	1.589 (0.41, 2.77)^a^
Upward	243 (17.2%)	0.514 (−0.93, 1.96)

^a^ *p* < 0.05; REF, Reference group; *n*(%), number of participants in each group (valid %) using the 3-group scale for financial status and transformed to show differences in mean CES-D scores.

### 3.5. Trajectory analysis

**Figure 1:**
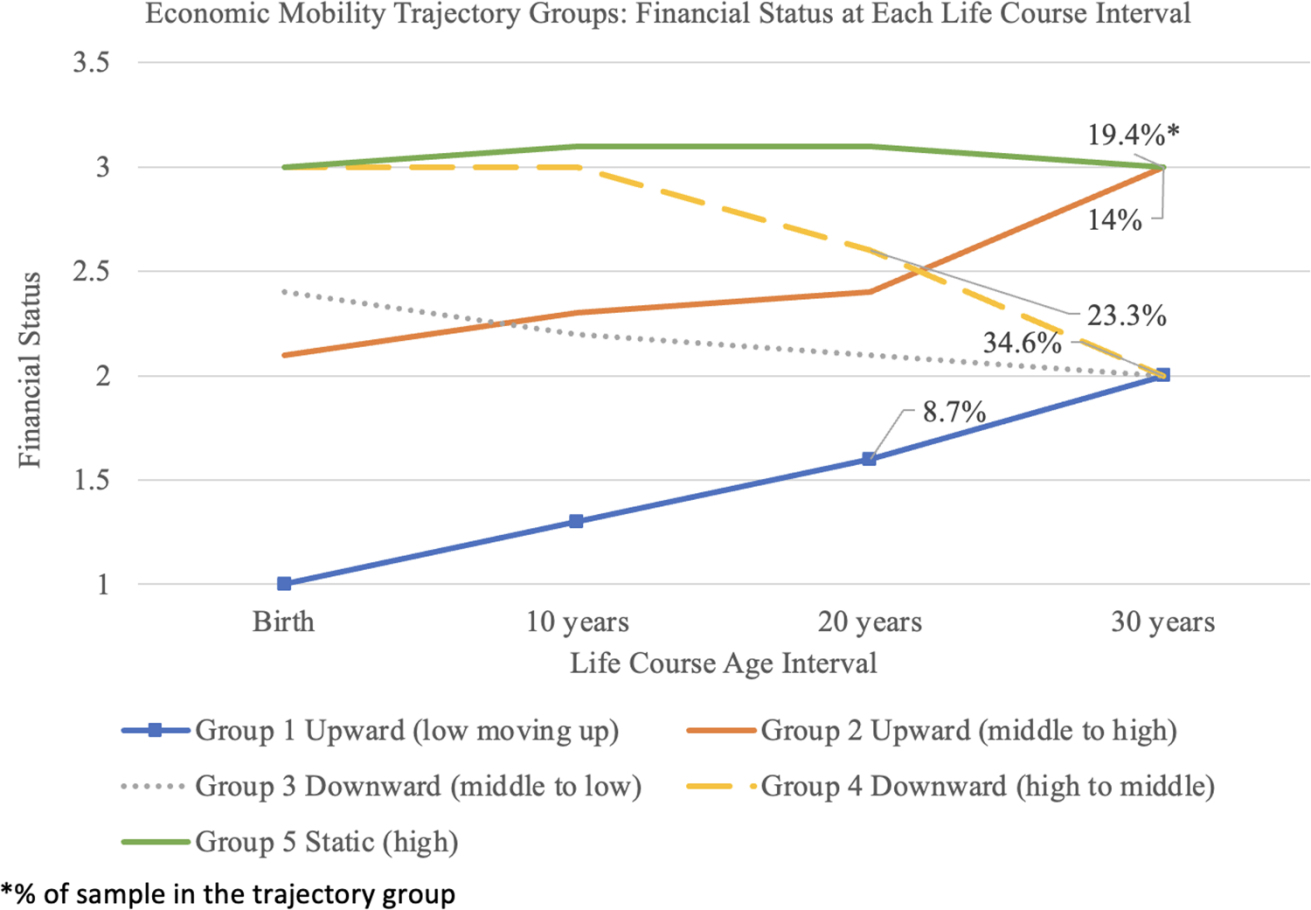
Economic Mobility Trajectory Groups: Financial Status at each Life Course Interval.

Five group trajectories of financial status over the life course were identified, as described in Figure 1: (1) Upward Low: *starting low, moving up*; (2) Upward Middle: *starting middle, moving up*; (3) Downward Middle: *starting middle, moving down*; (4) Downward High: *starting high, moving down*, and 5) Static: *no change*. Both the upward and downward social mobility groups have higher depression scores compared to the static (high) social mobility group when examining early childhood to current age in adulthood (Table 7). Further, the mean CES-D scores for upward and downward mobility groups are 16 or higher, indicating clinical depression, compared with the static group mean CESD score of 12.91.

**Table 7:** Linear regression: economic trajectory groups predicting CES-D score.

Groups	*N* (%)	Mean CES-D	Differences in Mean CES-D (95% CI)	Controlling for Maternal Age (95 CI)
Static: High (Group 5)	322 (22.8%)	12.91	REF	REF
Upward Low: Early Childhood low to middle (Group 1)	117 (8.3%)	17.41	4.23 (2.14, 6.32)^a^	4.51 (2.43, 6.6)^a^
Upward Middle: Early Childhood middle to high (Group 2)	236 (16.7%)	14.18	1.28 (0.37, 2.92)	1.43 (−0.21, 3.07)
Downward Middle: Early childhood middle to low (Group 3)	425 (30.1%)	16.89	3.99 (2.56, 5.41)^a^	4.04 (2.62, 5.46)^a^
Downward High: Early childhood high to middle (Group 4)	310 (22.8%)	15.97	2.96 (1.43, 4.5)^a^	3.09 (1.57, 4.62)^a^
Maternal Age	1,410 (100%)			−0.16 (−0.24, −0.08)^a^

^a^ *p* < 0.05; RE, Reference group; *n*(%), number of participants in each group (valid %) using the 3-group scale for financial status examining childhood to current age in adulthood.

## 4. Discussion

We examined how Black maternal psychological health in pregnancy can be understood in the context of the social mobility through the life course. Financial status, economic mobility, and depressive symptoms in pregnancy were examined from the women's perspective during pregnancy. This study highlights the impact of financial status, during childhood and currently, on depressive symptoms during pregnancy. Higher financial status was negatively associated with depressive symptoms. Both upward and downward mobility was associated with depressive symptoms for the preferred trajectory-based model during a woman's pregnancy.

Pregnancy is critical period in the life course. Maternal prenatal depression is associated with adverse birth outcomes (61) and childhood health outcomes (62, 63). The study results suggest that financial status, whether considered in early childhood, adolescence or adulthood predicts depressive symptoms among pregnant Black women. Further, upward and downward economic mobility was associated with higher CESD scores compared to those whose financial situation was static. The association between socioeconomic position and psychological well-being among pregnant Black women has been previously reported (29, 64). This study extends this research and provides additional context regarding the association of social mobility over the life course with depressive symptoms in pregnancy. This study provides new insight into the effects of perceived financial status changes over the life course on mental health of pregnant Black women. The results suggest that better financial status has a protective effect on the mental health during pregnancy, whereas both the positive and negative life-course financial status movements have a deleterious effect on the mental health of pregnant women. Current and childhood financial status has previously been shown to have an effect on mental health among adults (65–67). A study conducted by Kosidou et al. (68) showed that belonging to the highest household income category has a protective effect on depression in women. Similarly, this study supports that higher financial status has a protective effect against depressive symptoms.

During the early childhood and adolescence age intervals, the majority of the participants reported a better financial status than currently in adulthood and there was little economic movement between the early childhood and adolescent age intervals. Further, during the older age intervals, more mobility is observed in the downward financial mobility group. Most of the participants did not experience a movement in their financial status before 18 years of age. Trajectory analysis demonstrated that both upward and downward social mobility over the life had an adverse effect on the depressive symptoms of pregnant women. It is notable that the mean CES-D scores are higher in the upward mobility group. While controlling for confounding, we find the effect of age may be masking the effect of the upward mobility given this group has a higher proportion of younger women.

Black maternal health and mental health is increasingly recognized as a priority public health topic. In the current study, approximately 50% of the pregnant Black women reported CES-D score ≥16, which is correlated with clinically relevant depression, and higher than previous studies (64, 69). Further over 20% of the participants had scores >23, indicating high levels of depressive symptoms. This is higher than in other studies conducted with Black women (70) and may signify the importance of conducting studies and interventions to reduce the depression rates in this community, especially as preterm birth and infant mortality rates are higher among Black women. Given the established negative impact of depression on health and wellbeing, exploring how SEP may influence risk of depression across the life course is important. Additional research is needed in this area (54).

Higher SEP is generally associated with better health across the life course compared to those with lower SEP, often referred to as the health wealth gradient. However, recent studies have found that upward social mobility is associated with a tradeoff between well-being and health (28). Although the association of upward economic mobility with increased risk for depression in pregnancy is surprising, the minorities diminished return theory (22) may be a plausible explanation. The minorities diminished return theory suggests that education attainment and higher SEP offers a weaker protective effect for racial and ethnic minority groups that have been socially marginalized compared to non-Hispanic white individuals who are socially privileged. This pattern has been consistently supported by previous research across a wide range of physical health and mental health outcomes (e.g., self-rated health, cardiovascular disease, depression, obesity, and mortality) (22, 71, 72). There is also a growing body of literature that demonstrates higher education is associated with less health benefits for LGBT individuals than non-LGBT individuals. These differential benefits of SEP, social mobility, and health may be due, in part, to the upstream social processes and structure of US social systems that reduces the full benefit from mental health and financial resources available by increasing financial status (72). Research among Black women on social mobility and depressive symptoms is particularly important given the intersection of race and gender that may in turn increase risk of depressive symptoms.

### 4.1. Strengths & limitations

The LIFE study is strengthened by the large representative sample, use of a valid and reliable measure to assess depressive symptoms and focus on the social mobility of Black women across the three intervals over the life course. This study is innovative in its use of group trajectory analysis to examine the association between social mobility at three time intervals across the life course and depression, rather than using conventional indicators to measure social mobility at one time point (e.g., using summary scores of multiple individual indicators of SES).

There are also several methodological limitations to consider. Participants were recruited from a single suburban hospital in Metropolitan Detroit, Michigan. However, the study sample participants had similar demographic characteristics and birth outcomes as Black women in the United States for the study period. In addition, the hospital was selected for recruitment for several reasons: its wide catchment area, the heterogeneity of patients from 3 counties, and the large number of births per year. Perceived financial status was self-reported for the three-time intervals which may be influenced by recall bias and/or social desirability bias, particularly when reporting on the early childhood time interval. However, it is important to note the aim of the study is to understand the impact of *perceived* financial status on maternal health. Recent studies have suggested that multiple respondents may improve validity when examining life course factors (59) to fully capture experiences, particularly when multiple time periods are measured. The larger LIFE study does include interviews with the mother of the participant so future analysis will be able to include this additional information. One study has reported high agreement between participants and her mother on financial status in childhood (59). Data were collected during the first 24–72 h after birth. CES-D items measure depressive symptoms within the past week. Therefore, it is possible that women reported depressive symptoms experienced based on the outcome of birth rather than prenatal depressive symptoms. If women with depressive symptoms differentially report financial status, recall bias would be an issue.

Women may have had fluctuations in financial status within the interval periods which would not be captured by the three brackets defined by this study. More than 70% of the participants reported >12 years of education, which precluded further stratification in the lower-education group. Given the observational nature of the study, there exists the possibility of confounding due to unmeasured individual factors early in childhood that may influence both maternal health and SEP across the life course. Social mobility research is primarily interested in estimating variation in the effect of SEP and changes over time. Thus, overcontrol in analysis and selection biases in design can be consequential and are essential to consider. It is important to acknowledge that income is only one component of social mobility. Other relevant SEP measures (e.g., maternal occupation, previous occupation, education, etc) or information related to the fathers or paternal characteristics were not assessed. Hudson et al. (25) suggests that community-level economic factors, such as neighborhood resources, may be more informative in the association with depression rather than individual SEP factors.

Another limitation of this study is economic mobility is characterized as either upward or downward without considering magnitude of change. Further, movement within childhood or within adulthood are considered equivalent changes. This introduces two problems. Firstly, upward or downward movements of financial status between different strata may not have differential impacts. Secondly, upward and downward movements are grouped without consideration to the magnitude of change, which may have a role in association with depressive symptoms. Additionally, the static group is defined as no change in financial status and therefore includes those women who remain in the lower financial status as well as those who remain in higher financial status. Finally, approximately half of the women in the study were older, married, or cohabiting and had at least a high school education. The generalizability of the results may be limited to women with similar ethnic, socioeconomic, and residential characteristics. Future research is also necessary to replicate these findings in other contexts and settings to explore mediators and moderators of social mobility across the life course and depression among Black women, including measures of SEP.

### 4.2. Implications for policies to reduce maternal health disparities

Health inequalities cannot be addressed by narrowing SEP gap across social groups alone (73). There is a need for economic and health policies that examine the contextual, economic, and behavioral mediators of social mobility and depression in Black women, particularly in the critical period during pregnancy (74). Pregnancy offers an opportunity for women to engage with the health care system. There is growing recognition among health care providers that social determinants can be addressed in clinical settings. Medical education and health care providers are critical to advance health equity by incorporating the social determinants of health in practice (75). Given the consistent associations with high depressive symptoms in all levels of social mobility these results support the recommendation of providing mental health resources to Black women experiencing low SEP at any stage in life course. It is recommended to screen for depressive symptoms early in pregnancy and refer those who report depressive symptoms for treatment. Early identification to support women with depressive symptoms early in pregnancy has the potential to improve maternal mental health and birth outcomes. Maternal and child health programs that aim to improve financial status and mobility may also support mental health during pregnancy.

## Data Availability

The raw data supporting the conclusions of this article will be made available by the authors, without undue reservation.

## References

[1] Centers for Disease Control and Prevention. PRAMStat data portal. (2021). Available at: https://www.cdc.gov/prams/prams-data/mch-indicators/states/pdf/2020/All-Sites-PRAMS-MCH-Indicators-508.pdf (Accessed May 17, 2023).

[2] Giurgescu C Zenk SN Templin TN Giurgescu C Zenk SN Templin TN The impact of neighborhood environment, social support, and avoidance coping on depressive symptoms of pregnant African-American women. Womens Health Issues. (2015) 25(3):294–302. 10.1016/j.whi.2015.02.001 25840930 PMC4431761

[3] Dunkel Schetter C Tanner L. Anxiety, depression and stress in pregnancy implications for mothers, children, research, and practice. Curr Opin Psychiatry. (2012) 25(2):141–8. 10.1097/YCO.0b013e3283503680 22262028 PMC4447112

[4] Woody CA Ferraria AJ Siskin DJ Whiteford HA Harris MG. A systematic review and meta-regression of the prevalence and incidence of perinatal depression. J Affect Disord. (2017) 219:86–92. 10.1016/j.jad.2017.05.003 28531848

[5] Davalos D Yadon C Tregellas H. Untreated prenatal maternal depression and the potential risks to offspring: a review. Arch Women's Mental Health. (2012) 15(1):1–14. 10.1007/s00737-011-0251-1 22215285

[6] Grote NK Bridge JA Gavin AR Melville JL Iyengar S Katon WJ. A meta-analysis of depression during pregnancy and the risk of preterm birth, low birth weight, and intrauterine growth restriction. Arch Gen Psychiatry. (2010) 67(10):1012–24. 10.1001/archgenpsychiatry.2010.111 20921117 PMC3025772

[7] Weobong B Ten Asbroek AH Soremekun S Manu AA Owusu-Agyei S Prince M Association of antenatal depression with adverse consequences for the mother and newborn in rural Ghana: findings from the DON population-based cohort study. PLoS One. (2014) 9(12):e116333. 10.1371/journal.pone.0116333 25549334 PMC4280205

[8] Brody DJ Pratt LA Hughes JP. Prevalence of depression among adults aged 20 and over: United States, 2013–2016. NCHS Data Brief. (2018) 303:1–8. https://www.cdc.gov/nchs/products/databriefs/db303.htm 29638213

[9] National Institute of Mental Health. Depression. (NIH publication No. 21-MH-8079). Bethesda, MD: U.S. Department of Health and Human Services, National Institutes of Health (2021).

[10] Glymour M Avendano M Kawachi I. Socioeconomic status and health. In: Berkman LF Kawachi I Glymour M, editors. Social epidemiology. 2nd ed. Oxford: Oxford University Press (2014). p. 17–62.

[11] Blumenshine P Egerter S Barclay CJ Cubbin C Braveman PA. Socioeconomic disparities in adverse birth outcomes: a systematic review. Am J Prev Med. (2010) 39(3):263–72. 10.1016/j.amepre.2010.05.012 20709259

[12] Mukherjee S Trepka M Pierre-Victor D Behelah R Avent T. Racial/ethnic disparities in antenatal depression in the United States: a systematic review. Matern Child Health J. (2016) 20(9):1780–97. 10.1007/s10995-016-1989-x 27016352

[13] Ko JY Farr SL Dietz PM Robbins CL. Depression and treatment among U.S. pregnant and nonpregnant women of reproductive age, 2005-2009. J Womens Health. (2012) 21(8):830–6. 10.1089/jwh.2011.3466 PMC4416220 22691031

[14] Toffol E Koponen P Partonen T. Miscarriage and mental health: results of two population-based studies. Psychiatry Res. (2013) 205(1–2):151–8. 10.1016/j.psychres.2012.08.029 22985545

[15] Hawkins M Misra D Zhang L Price M Dailey R Giurgescu C. Family involvement in pregnancy and psychological health among pregnant Black women. Arch Psychiatr Nurs. (2021) 35(1):42–8. 10.1016/j.apnu.2020.09.012 33593514 PMC7890047

[16] Lorch SA Enlow E. The role of social determinants in explaining racial/ethnic disparities in perinatal outcomes. Pediatr Res. (2016) 79(1-2):141–7. 10.1038/pr.2015.199 26466077 PMC11836890

[17] Misra DP Slaughter-Acey J Giurgescu C Sealy-Jefferson S Nowak A. Why do Black women experience higher rates of preterm birth? Curr Epidemiol Rep. (2017) 4:83–97. 10.1007/s40471-017-0102-3

[18] Patel OP Quist A Martin CL Wegienka G Baird DD Wise LA Life-course mobility in socioeconomic position and high depressive symptoms among young Black women: the SELF study. Womens Health Issues. (2023) 33(3):266–72. 10.1016/j.whi.2022.11.010 36588050 PMC10213084

[19] Colen CG Krueger PM Boettner BL. Do rising tides lift all boats? Racial disparities in health across the lifecourse among middle-class African-Americans and whites. SSM Popul Health. (2018) 6:125–35. 10.1016/j.ssmph.2018.07.004 30258971 PMC6153271

[20] Colen CG Ramey DM Cooksey EC Williams DR. Racial disparities in health among nonpoor African Americans and Hispanics: the role of acute and chronic discrimination. Soc Sci Med. (2018) 199:167–80. 10.1016/j.socscimed.2017.04.051 28571900 PMC5673593

[21] Curry Owens T Jackson FM. Examining life-course socioeconomic position, contextualized stress, and depression among well-educated African-American pregnant women. Womens Health Issues. (2015) 25(4):382–9. 10.1016/j.whi.2015.05.001 26143076

[22] Assari S. Health disparities due to diminished return among Black Americans: public policy solutions. Soc Issues Policy Rev. (2018) 12(1):112–45. 10.1111/sipr.12042

[23] Hudson DL Neighbors HW Geronimus AT Jackson JS. Racial discrimination, John Henryism, and depression among African Americans. J Black Psychol. (2016) 42(3):221–43. 10.1177/0095798414567757 27529626 PMC4903152

[24] Budhwani H Hearld KR Chavez-Yenter D. Depression in racial and ethnic minorities: the impact of nativity and discrimination. J Racial Ethn Health Disparities. (2015) 2(1):34–42. 10.1007/s40615-014-0045-z 26863239

[25] Hudson DL Puterman E Bibbins-Domingo K Matthews KA Adler NE. Race, life course socioeconomic position, racial discrimination, depressive symptoms and self-rated health. Soc Sci Med. (2013) 97:7–14. 10.1016/j.socscimed.2013.07.031 24161083

[26] Joinson C Kounali D Lewis G. Family socioeconomic position in early life and onset of depressive symptoms and depression: a prospective cohort study. Soc Psychiatry Psychiatr Epidemiol. (2017) 52(1):95–103. 10.1007/s00127-016-1308-2 27837235 PMC5226994

[27] Ruiz M Hu Y Martikainen P Bobak M. Life course socioeconomic position and incidence of mid-late life depression in China and England: a comparative analysis of CHARLS and ELSA. J Epidemiol Community Health. (2019) 73(9):817–24. 10.1136/jech-2019-212216 31255999

[28] Miller GE Chen E Yu T Brody GH. Youth who achieve upward socioeconomic mobility display lower psychological distress but higher metabolic syndrome rates as adults: prospective evidence from Add Health and MIDUS. J Am Heart Assoc. (2020) 9(9):e015698. 10.1161/JAHA.119.015698 32340532 PMC7428555

[29] Slaughter-Acey JC Holzman C Calloway D Tian Y. Movin’ on up: socioeconomic mobility and the risk of delivering a small-for-gestational age infant. Matern Child Health J. (2016) 20(3):613–22. 10.1007/s10995-015-1860-5 26541591 PMC4754152

[30] Morrissey K Kinderman P. The impact of childhood socioeconomic status on depression and anxiety in adult life: testing the accumulation, critical period and social mobility hypotheses. Population Health. (2020) 11:100576. 10.1016/j.ssmph.2020.100576 PMC7178545 32346597

[31] Verropoulou G Serafetinidou E Tsimbos C. Decomposing the effects of childhood adversity on later-life depression among Europeans: a comparative analysis by gender. Ageing Soc. (2021) 41(1):158–86. 10.1017/S0144686X19000977

[32] McLean CP Asnaani A Litz BT Hofmann SG. Gender differences in anxiety disorders: prevalence, course of illness, comorbidity and burden of illness. J Psychiatr Res. (2011) 45(8):1027–35. 10.1016/j.jpsychires.2011.03.006 21439576 PMC3135672

[33] St Clair MC Croudace T Dunn VJ Jones PB Herbert J Goodyer IM. Childhood adversity subtypes and depressive symptoms in early and late adolescence. Dev Psychopathol. (2015) 27(3):885–99. 10.1017/S0954579414000625 25058564 PMC4531475

[34] Verropoulou G Serafetinidou E. Childhood and adulthood circumstances predicting affective suffering and motivation among older adults: a comparative study of European welfare systems. Eur J Ageing. (2019) 16(4):425–38. 10.1007/s10433-019-00518-w 31798368 PMC6857123

[35] Angelini V Howdon DD Mierau JO. Childhood socioeconomic status and late-adulthood mental health: results from the survey on health, ageing and retirement in Europe. J Gerontol B. (2019) 74(1):95–104. 10.1093/geronb/gby028 PMC6941210 29566242

[36] Reeves R Krause E. The effects of maternal depression on early childhood development and implications for economic mobility. Washington, DC: Brookings Institute (2019). Available at: https://www.brookings.edu/wp-content/uploads/2019/01/ES_20190131_Reeves_Maternal_Depression2.pdf (Accessed May 17, 2023).

[37] Novak M Ahlgren C Hammarstrom A. Social and health-related correlates of intergenerational and intragenerational social mobility among Swedish men and women. Public Health. (2012) 126(4):349–57. 10.1016/j.puhe.2012.01.012 22444320

[38] Nicklett EJ Burgard SA. Downward social mobility and major depressive episodes among Latino and Asian-American immigrants to the United States. Am J Epidemiol. (2009) 170(6):793–801. 10.1093/aje/kwp192 19671834 PMC2768522

[39] Corak M. Income inequality, equality of opportunity, and intergenerational mobility. J Econ Perspect. (2013) 27(3):79–102. 10.1257/jep.27.3.79

[40] James SA Fowler-Brown A Raghunathan TE Van Hoewyk J. Life-course socioeconomic position and obesity in Black women: the Pitt county study. Am J Public Health. (2006a) 96(3):554–60. 10.2105/ajph.2004.053447 16449599 PMC1470506

[41] James SA Van Hoewyk J Belli RF Strogatz DS Williams DR Raghunathan TE. Life-course socioeconomic position and hypertension in Black men: the Pitt county study. Am J Public Health. (2006b) 96(5):812–7. 10.2105/ajph.2005.076158 16571689 PMC1470586

[42] Maty SC James SA Kaplan GA. Life-course socioeconomic position and incidence of diabetes mellitus among Blacks and whites: the Alameda county study, 1965–1999. Am J Public Health. (2010) 100(1):137–45. 10.2105/ajph.2008.133892 19197084 PMC2791248

[43] Hoven H Siegrist J Goldberg M Ribet C Zins M Wahrendorf M. Intragenerational social mobility and depressive symptoms. Results from the French CONSTANCES cohort study. SSM Popul Health. (2019) 7:100351. 10.1016/j.ssmph.2019.100351 30705934 PMC6349560

[44] Misra DP Guyer B Allston A. Integrated perinatal health framework: a multiple determinants model with a life span approach. Am J Prev Med. (2003) 25(1):65–75. 10.1016/S0749-3797(03)00090-4 12818312

[45] Linander I Hammarström A Johansson K. Which socio-economic measures are associated with psychological distress for men and women? A cohort analysis. Eur J Public Health. (2015) 25(2):231–6. 10.1093/eurpub/cku137 25172836

[46] Osypuk T Slaughter-Acey J Kehm R Misra DP. Life course social mobility reduces risk of adverse birth outcomes. Am J Prev Med. (2016) 51(6):975–82. 10.1016/j.amepre.2016.09.008 27866597 PMC5167500

[47] Chetty R Hendren N. The impacts of neighborhoods on intergenerational mobility II: county-level estimates. Q J Econ. (2018) 133:1163–228. 10.1093/qje/qjy006

[48] Colen CG Geronimus AT Bound J James SA. Maternal upward socioeconomic mobility and black-white disparities in infant birthweight. Am J Public Health. (2006) 96(11):2032–9. 10.2105/ajph.2005.076547 17018818 PMC1751798

[49] Hallqvist J Lynch J Bartley M Lang T Blane D. Can we disentangle life course processes of accumulation, critical period and social mobility? An analysis of disadvantaged socioeconomic positions and myocardial infarction in the Stockholm heart epidemiology program. Soc Sci Med. (2004) 58(8):1555–62. 10.1016/S0277-9536(03)00344-7 14759698

[50] Lacey RE Gondek D Smith BJ Smith ADAC Dunn EC Sacker A. Testing lifecourse theories characterising associations between maternal depression and offspring depression in emerging adulthood: the Avon longitudinal study of parents and children. J Child Psychol Psychiatry. (2022) 64(8):10.1111/jcpp.13699. 10.1111/jcpp.13699 PMC10008452 36094018

[51] Lu W Liu N Chen J. Subjective social mobility among migrant children in China. Int J Environ Res Public Health. (2022) 19(9):5685. 10.3390/ijerph19095685 35565080 PMC9104079

[52] Pudrovska T Anikputa B. Early-life socioeconomic status and mortality in later life: an integration of four life-course mechanisms. J Gerontol B Psychol Sci Soc Sci. (2014) 69(3):451–60. 10.1093/geronb/gbt122 24496607 PMC3983914

[53] Sealy-Jefferson S Giurgescu C Helmkamp L Misra DP Osypuk TL. Perceived physical and social residential environment and preterm delivery in African-American women. Am J Epidemiol. (2015) 182(6):485–93. 10.1093/aje/kwv106 26163532 PMC4668760

[54] Giurgescu C Misra DP Sealy-Jefferson S Caldwell CH Templin TN Slaughter- Acey JC The impact of neighborhood quality, perceived stress, and social support on depressive symptoms during pregnancy in African American women. Soc Sci Med. (2015) 130:172–80. 10.1016/j.socscimed.2015.02.006 25703670 PMC4431774

[55] Radloff LS. The CES-D scale: a self report depression scale for research in general population. Appl Psychol Meas. (1977) 1(3):385–401. 10.1177/014662167700100306

[56] Atkins R. Validation of the center for epidemiologic studies depression scale in Black single mothers. J Nurs Meas. (2014) 22(3):511–24. 10.1891/1061-3749.22.3.511 25608436 PMC4389584

[57] Orr ST Orr CA James SA Blazer DG. Life satisfaction and preterm birth among urban Black women: findings from the Baltimore preterm birth study. Ann Epidemiol. (2012) 22(11):759–63. 10.1016/j.annepidem.2012.09.002 23041653

[58] Jackson JS Torres M Caldwell CH Neighbors HW Nesse RM Taylor RJ The national survey of American life: a study of racial, ethnic and cultural influences on mental disorders and mental health. Int J Methods Psychiatr Res. (2004) 13(4):196–207. 10.1002/mpr.177 15719528 PMC6878295

[59] Straughen JK Caldwell CH Osypuk TL Helmkamp L Misra DP. Direct and proxy recall of childhood socio-economic position and health. Paediatr Perinat Epidemiol. (2013) 27(3):294–302. 10.1111/ppe.12045 23574418 PMC3637926

[60] Radloff LS Locke B. The community mental health assessmrnt survey and CES-D scale. New Brunswick, NJ: Rutgers University Press (1986).

[61] Accortt EE Cheadle AC Dunkel Schetter C. Prenatal depression and adverse birth outcomes: an updated systematic review. Matern Child Health J. (2015) 19(6):1306–37. 10.1007/s10995-014-1637-2 25452215 PMC4447551

[62] Silva CCV Vehmeijer FOL El Marroun H Felix JF Jaddoe VWV Santos S. Maternal psychological distress during pregnancy and childhood cardio-metabolic risk factors. Nutr Metab Cardiovasc Dis. (2019) 29(6):572–9. 10.1016/j.numecd.2019.02.008 30956027

[63] Vehmeijer FOL Guxens M Duijts L El Marroun H. Maternal psychological distress during pregnancy and childhood health outcomes: a narrative review. J Dev Orig Health Dis. (2019) 10(3):274–85. 10.1017/S2040174418000557 30378522

[64] Giurgescu C Templin TN. Father involvement and psychological well-being of pregnant women. MCN Am J Matern Child Nurs. (2015) 40(6):381–7. 10.1097/NMC.0000000000000183 26488855 PMC4617560

[65] Nicholson A Pikhart H Pajak A Malyutina S Kubinova R Peasey A Socio-economic status over the life-course and depressive symptoms in men and women in Eastern Europe. J Affect Disord. (2008) 105(1-3):125–36. 10.1016/j.jad.2007.04.026 17561267

[66] Power C Atherton K Strachan DP Shepherd P Fuller E Davis A Life-course influences on health in British adults: effects of socio-economic position in childhood and adulthood. Int J Epidemiol. (2007) 36(3):532–9. 10.1093/ije/dyl310 17255345

[67] Stansfeld SA Clark C Rodgers B Caldwell T Power C. Repeated exposure to socioeconomic disadvantage and health selection as life course pathways to mid-life depressive and anxiety disorders. Soc Psychiatry Psychiatr Epidemiol. (2011) 46(7):549–58. 10.1007/s00127-010-0221-3 20383489 PMC3112323

[68] Kosidou K Dalman C Lundberg M Hallqvist J Isacsson G Magnusson C. Socioeconomic status and risk of psychological distress and depression in the Stockholm public health cohort: a population-based study. J Affect Disord. (2011) 134(1-3):160–7. 10.1016/j.jad.2011.05.024 21665286

[69] Phillips GS Wise LA Rich-Edwards JW Stampfer MJ Rosenberg L. Prepregnancy depressive symptoms and preterm birth in the black Women's Health study. Ann Epidemiol. (2010) 20(1):8–15. 10.1016/j.annepidem.2009.09.009 20006271 PMC2796255

[70] Orr ST James SA Blackmore Prince C. Maternal prenatal depressive symptoms and spontaneous preterm births among Black women in Baltimore, Maryland. Am J Epidemiol. (2002) 156(9):797–802. 10.1093/aje/kwf131 12396996

[71] Assari S. Unequal gain of equal resources across racial groups. Int J Health Policy Manag. (2017) 7(1):1–9. 10.15171/ijhpm.2017.90 PMC5745862 29325397

[72] Assari S Cobb S Cuevas AG Bazargan M. Diminished health returns of educational attainment among immigrant adults in the United States. Front. Psychiatry. (2020) 11:535624. 10.3389/fpsyt.2020.535624 33329080 PMC7728619

[73] Assari S. Family income reduces risk of obesity for white but not Black children. Children. (2018) 5(6):73–86. 10.3390/children5060073 29890778 PMC6025246

[74] Jones NL Gilman SE Cheng TL Drury SS Hill CV Geronimus AT. Life course approaches to the causes of health disparities. Am J Public Health. (2019) 109(S1):S48–55. 10.2105/AJPH.2018.304738 30699022 PMC6356123

[75] Rao R Hawkins M Ulrich T Gatlin G Mabry G Mishra C. The evolving role of public health in medical education. Front Public Health. (2020) 8:251. 10.3389/fpubh.2020.00251 32714890 PMC7344251

