# Corrigendum: Social mobility and perinatal depression in Black women

**DOI:** 10.3389/frhs.2024.1349547

**Published:** 2024-01-24

**Authors:** Melissa Hawkins, Arun Mallapareddi, Dawn P. Misra

**Affiliations:** ^1^ Department of Health Studies, College of Arts and Sciences, American University, Washington, DC, United States; ^2^ Department of Family Medicine and Public Health Sciences, School of Medicine, Wayne State University, Detroit, MI, United States

**Keywords:** social mobility, economic mobility, mental health, pregnancy, black, depression, CES-D

A Corrigendum on Social mobility and perinatal depression in Black women By Hawkins M, Mallapareddi A and Misra D (2023) Front. Health Serv. 3:1227874. doi: 10.3389/frhs.2023.1227874

**Incorrect funding**

In the published article, there was an error in the Funding statement. The funder and grant number were not included in the Funding Information Statement of the original article. The following statements regarding funding were originally provided: none.

The correct Funding statement appears below.

**FUNDING**

This study received funding from National Institutes of Health (NIH) grant no. R01HD058510. The funder was not involved in the study design, collection, analysis, interpretation of data, the writing of this article or the decision to submit it for publication. All authors declare no other competing interests.

The authors apologize for this error and state that this does not change the scientific conclusions of the article in any way. The original article has been updated.

