# Supplementary material for: Social mobility and perinatal depression in Black women
Source: Front Health Serv. 2023 Aug 24;3:1227874. doi: 10.3389/frhs.2023.1227874 (PMC10491480; doi:10.3389/frhs.2023.1227874)
Supplement: Supplementary file 1 [file Datasheet1.doc]

S1. INTERVIEW ID # ____

FOR OFFICE USE ONLY

FAMILY # ____

Interview: #1

**RISK FACTORS FOR PRETERM BIRTH IN BLACK WOMEN**

***MOTHERS’* QUESTIONNAIRE**

| 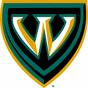  1  Wayne State University | S2. IWER ID: _________  S3. SITE: __________ |
| --- | --- |

S4. Date IW Taken: ___ ___/___ ___/___ ___

S5. Length of IW: ______ (minutes)

| INTRODUCTION  Hello, my name is ________ and I am from Wayne State University. We are currently conducting a study that will help us find out more about why some women go into labor earlier than other women. This is important to know because babies who are born too early may have more problems than other babies. We are inviting all African American women who are having a baby here at Providence Hospital to be in this study, even if a woman did not have any problems with her pregnancy. I was hoping that today you would be willing to answer some questions to assist us in our research.    This interview is completely voluntary and private -- if we should come to any question you don't want to answer, please tell me and we will go on to the next question.  I think you'll find the questions interesting. |
| --- |

Wayne State University, January 11, 2011

**Consent check-off and Med Record information**

**(DID YOU READ AND REVIEW THE CONSENT FORM WITH R?) 1. YES 5. NO**

**(DID R SIGN AND RETURN CONSENT FORM? 1. YES 5. NO GIVE R COPY OF**

**CONSENT FORM TO SIGN**

**R’s DOB from Medical Record (use format 01/01/1900):** _____________________

**R’s current address from Medical Record**

Apartment or House Number: _____________________________

Street: _______________________________________________

City: _____________________________________________________

State: _____________________________________________________

Zip: _____________________________________________________

Phone number #1: _________________________________________

Phone number #2: _________________________________________

**HOUSEHOLD LISTING**

**EXACT TIME NOW: ________**

First, I need to list all of the people you live with. This is just to get an idea of what your household is like. I don’t need details, just enough information to describe the different living arrangements of study participants as a group. Let me assure you that everything you tell me will be kept confidential.

Please tell me the first name and last initial, sex, age, and relationship to you of everybody who lives with you now. **[If child of R, write information in table on page 3]**

|  | A. | B. | C. | D. |
| --- | --- | --- | --- | --- |
|  | Name (First & Last Initial) for Each Household Member | Household Member's  Relationship to Respondent | Sex | Age |
| **T1** |  |  | **F** |  |
| **T2** |  |  | **F** |  |
| **T3** |  |  | **F** |  |
| **T4** |  |  | **F** |  |
| **T5** |  |  | **F** |  |
| **T6** |  |  | **F** |  |
| **T7** |  |  | **F** |  |
| **T8** |  |  | **M** |  |
| **T9** |  |  | **M** |  |
| **T10** |  |  | **M** |  |
| **T11** |  |  | **M** |  |
| **T12** |  |  | **M** |  |

You have said that (REPEAT NAME LISTING) live with you. Does that include everyone living with you now? (IF NO, CORRECT THE LISTING.)

Please tell me the name, sex and age of each of your children.

|  | A. | B. | C. | D. |
| --- | --- | --- | --- | --- |
|  | Check Here if R  Volunteers that  Child is No Longer  Living | First Name and Last Initial  For Each Child Mentioned | Sex | Age |
| **T13** |  |  | **F** |  |
| **T14** |  |  | **F** |  |
| **T15** |  |  | **F** |  |
| **T16** |  |  | **F** |  |
| **T17** |  |  | **F** |  |
| **T18** |  |  | **F** |  |
| **T19** |  |  | **F** |  |
| **T20** |  |  | **M** |  |
| **T21** |  |  | **M** |  |
| **T22** |  |  | **M** |  |
| **T23** |  |  | **M** |  |
| **T24** |  |  | **M** |  |

INTERVIEWER CHECKPOINT

| COUNT AND RECORD TOTAL NUMBER OF CHILDREN ENTERED ON DIAGRAM ABOVE.  **T25.** CHILDREN _____ |
| --- |
|  |

**SECTION A: FAMILY STRUCTURE AND ECONOMICS**

A1. To begin, I would like to know a little about you and your family. Could you please tell me where you were born?

____________________________________________ **(IF U.S., GO TO A2)**

CITY/STATE/COUNTRY

A1a. What year did you come to the US? ___________________________________

**NOTE TO INTERVIEWER: IF R ANSWERS ‘SKIP’ OR ‘DON’T KNOW’ TO ANY OF THE FOLLOWING QUESTIONS WITHOUT A SKIP OR DON’T KNOW OPTION TO CIRCLE, PLEASE WRITE SKIP OR DON’T KNOW NEXT TO THE QUESTION.**

A2. Are you currently married or living with a partner, divorced, separated, or have you never married?

1. Married

2. Living with Partner

3. Widowed

4. Divorced

5. Separated

6. Never Married

**GO TO A2c** **GO TO A2d**

A2a. Are you married to/living with (father of baby)?

1. YES

5. NO

A2b. How long have you been married/living with partner? __________ **Go To A2g**

A2c. How long have you been (response to A2)? _________

A2d. Are you currently going with anyone?

1. YES

5. NO

**Go to A2g**

A2e. Is that person (father of baby)?

1. YES

5. NO

**Go to A2h**

A2f. How long have you been going with (father of baby)? ____________

A2g. How long have you known (father of baby/FOB)? ____________ **Go to A2j**

A2h. How long have you been going with (referent in A2d)? ____________

A2i. How long have you known (father of baby/FOB)? ____________

| A2j. Where is the (FOB) currently living?  ***IF NECESSARY, USE ANSWER CATEGORIES TO PROBE*** | Apartment in a House or Garden Apartment (1-4 stories) ……….…..01  High Rise Apartment (more than 4 floors) ………………………………... 02  Rowhouse or Duplex…………………………………………………………03  Detached Single Family House……………………………………………..04  Shelter ………………………………………………………….…05  Correctional Facility, Institution or Other Kind of Group Arrangement… 06  Specify: ___________________________________________________  Other…………………………………………………………………………...07  Specify: ___________________________________________________ | A2j.__ |
| --- | --- | --- |

A3. **(HAND R YELLOW CARD SIDE 1)** How would you describe your family's financial situation today? Would you say ...

1. VERY POOR, NOT ENOUGH TO GET BY

2. BARELY ENOUGH TO GET BY

3. HAVE ENOUGH TO GET BY BUT NO EXTRAS

4. HAVE MORE THAN ENOUGH TO GET BY

5. WELL TO DO

7. SKIPPED

9. DON’T KNOW

A4. **(YELLOW CARD SIDE 1)** How would you describe your family's financial situation while you were growing up? Would you say...

7. SKIPPED

1. VERY POOR, NOT ENOUGH TO GET BY

2. BARELY ENOUGH TO GET BY

3. HAD ENOUGH TO GET BY BUT NO EXTRAS

4. HAD MORE THAN ENOUGH TO GET BY

5. WELL TO DO

9. DON’T KNOW

A4a. **(YELLOW CARD SIDE 1)** How would you describe your family's financial situation between the time you were born (birth) and your 10^th^ birthday? Would you say...

7. SKIPPED

1. VERY POOR, NOT ENOUGH TO GET BY

2. BARELY ENOUGH TO GET BY

3. HAD ENOUGH TO GET BY BUT NO EXTRAS

4. HAD MORE THAN ENOUGH TO GET BY

5. WELL TO DO

9. DON’T KNOW

A4b. **(YELLOW CARD SIDE 1)** How would you describe your family's financial situation between your 10^th^ and 18^th^ birthdays? Would you say...

1. VERY POOR, NOT ENOUGH TO GET BY

2. BARELY ENOUGH TO GET BY

3. HAD ENOUGH TO GET BY BUT NO EXTRAS

4. HAD MORE THAN ENOUGH TO GET BY

5. WELL TO DO

7. SKIPPED

9. DON’T KNOW

A5. Who makes a financial contribution to your household income? **(DO NOT READ CATEGORIES AND CHECK ALL THAT APPLY)**

1. SELF

2. FATHER OF BABY

3. LIVE-IN PARTNER

4. SPOUSE

5. YOUR FATHER/ MOTHER

6. OTHER (PLEASE SPECIFY RELATIONSHIP)

________________________________

A6. Are there other sources of income to your household?

1. YES

5. NO

**GO TO A7**

A6a. What are these? **[CHECK ALL THAT APPLY]**

1. SSI

2. WELFARE

3. UNEMPLOYMENT

4. FOOD STAMPS

5. ALIMONY

6. WIC

7. OTHER (PLEASE SPECIFY)

____________________________________________________________________________________________________________________________________

A7. Are there people who do not live with you who you help to support financially?

1. YES

5. NO

**GO TO A8**

A7a. How many? __________

A7b. Who are they? **[DO NOT READ CATEGORIES AND CHECK ALL THAT APPLY]**

1. MY BABY

2. ANOTHER CHILD

3. R’S MOTHER

4. FOB

5. MORE THAN ONE CHILD

6. R’S FATHER

7. OTHER (PLEASE SPECIFY)

____________________________________________________________________________________________________________________________________

A8. Who is the main financial provider in your household?

______________________________________________

A9. **(HAND R YELLOW CARD SIDE 2)** Please look at the **YELLOW CARD SIDE 2.** On a scale of 1 to 7, with 1 being completely satisfied and 7 being completely dissatisfied, all things considered, how satisfied are you with life as a whole these days? Would you say...

DK

SKIP

**1 2 3 4 5 6 7**

COMPLETELY NEITHER COMPLETELY

SATISFIED SATISFIED NOR DISSATISFIED

DISSATISFIED

**SECTION B: WORK STATUS**

B1. Are you currently working or temporarily laid-off from a regular job?

1. YES, WORKING

2. YES, TEMPORARY LAID OFF

3. NO

**GO TO B2**

B1a. Are you unemployed and looking for work, at home and not looking for work, a full-time student or what?

3. UNEMPLOYED

7. OTHER (PLEASE SPECIFY):

_______________________________________________________________

6. FULL-TIME STUDENT

5. AT HOME

4. RETIRED

**GO TO B7**  **GO TO B1b GO TO B7**

**GO TO B7**

B2. What sort of work do (did) you do?

B3. About how many hours do (did) you work on your job in an average week? _______________

(# HRS/WK)

| **GO TO B4 ON NEXT PAGE** |
| --- |

| **R IS UNEMPLOYED, AT HOME, FULL-TIME STUDENT, OR OTHER AT B1a** |
| --- |

B7. Have you ever worked for pay?

1. YES

5. NO

**GO TO SECTION C**

B8. How long has it been since you were last employed?

________MONTHS OR _______YEARS (data entry in months)

B9. What kind of work did you do on your last regular job?

______________________________________________________________________

______________________________________________________________________

B10. About how many hours did you work on your last job in an average week?

_________HRS/WK

B4. How steady is (was) your work? Would you say… ***(Preterm Delivery Study Sequence)***

1. REGULAR & STEADY

2. SEASONAL

3. FREQUENT LAYOFFS

4. OTHER: (Specify _______________________________

B5. **(HAND R RED CARD SIDE 1)** In the past year, *how often* were you in a situation where you faced job loss or layoff?

1. MOST OF THE TIME

2. ABOUT HALF THE TIME

3. SOME OF THE TIME

4. NEVER

B11. **INTERVIEWER CHECKPOINT**

SEE B3 AND B10

1. R HAS WORKED ≥ 30 HOURS A WEEK

2. ALL OTHERS **GO TO SECTION C**

***(Teen Fathers Study)***

B13. **(HAND R YELLOW CARD SIDE 2)** Please look at this card. On a scale of 1 to 7, with 1 being completely satisfied and 7 being completely dissatisfied, all things considered, how satisfied are you with your job?

**1 2 3 4 5 6 7**

DK

SKIP

COMPLETELY NEITHER COMPLETELY

SATISFIED SATISFIED NOR DISSATISFIED

DISSATISFIED

B14. **(YELLOW CARD SIDE 2)** How would you feel if a daughter of yours had your job as a regular, permanent job? Would you feel...

DK

SKIP

**1 2 3 4 5 6 7**

COMPLETELY NEITHER COMPLETELY

SATISFIED SATISFIED NOR DISSATISFIED

DISSATISFIED

**SECTION C: SOCIAL RELATIONSHIPS**

Now I'd like to ask you some questions about your family and friends.

C1. Please look at the **YELLOW CARD** **SIDE 2** again. On a scale of 1-7, how satisfied are you with your family relationships, that is, the things you do with your family and how you get along with them?

DK

SKIP

**1 2 3 4 5 6 7**

COMPLETELY NEITHER COMPLETELY

SATISFIED SATISFIED NOR DISSATISFIED

DISSATISFIED

C1a. How many close relatives (family members) do you have? ­­­____________ (number)_

__

C2. Do you have people that you consider to be your friends?

1. YES

5. NO

**GO TO C5**

C3. **(YELLOW CARD SIDE 2)** How satisfied are you with your friendships, that is, the things you do with your friends and how you get along with them?

DK

SKIP

**1 2 3 4 5 6 7**

COMPLETELY NEITHER COMPLETELY

SATISFIED SATISFIED NOR DISSATISFIED

DISSATISFIED

C3a. How many close friends do you have? ________ __ (number)_

C4. In general, would you say you receive more support from friends, family, or about the same amount of support from both?

1. FRIENDS

2. BOTH

3. FAMILY

7. SKIP

9. DK

***General Social Support – Preterm Delivery Study***

C5. People sometimes look to others for companionship, assistance, or other types of support. How often is each of the following kinds of support available to you if you need it?  ***(HAND R PURPLE CARD SIDE 1)*** Is it none of the time, a little of the time, some of the time, most of the time or all of the time?

|  | None of  the Time | A Little  of the Time | Some of  the Time | Most of  the Time | All of  the Time | Skip | Don’t Know |
| --- | --- | --- | --- | --- | --- | --- | --- |
| 1. Someone to help you if you were confined to bed | 1 | 2 | 3 | 4 | 5 | 7 | 9 |
| 1. Someone you can count on to listen when you need to talk | 1 | 2 | 3 | 4 | 5 | 7 | 9 |
| 1. Someone to give you good advice about a crisis… | 1 | 2 | 3 | 4 | 5 | 7 | 9 |
| 1. Someone to take you to the doctor if you needed it | 1 | 2 | 3 | 4 | 5 | 7 | 9 |
| 1. Someone who shows you love and affection | 1 | 2 | 3 | 4 | 5 | 7 | 9 |
| 1. Someone to have a good time with | 1 | 2 | 3 | 4 | 5 | 7 | 9 |
| 1. Someone to give you information to help you understand a situation | 1 | 2 | 3 | 4 | 5 | 7 | 9 |
| 1. Someone to confide in or talk to about yourself or your problems | 1 | 2 | 3 | 4 | 5 | 7 | 9 |
| 1. Someone who hugs you | 1 | 2 | 3 | 4 | 5 | 7 | 9 |
| 1. Someone to get together with for relaxation | 1 | 2 | 3 | 4 | 5 | 7 | 9 |
| 1. Someone to prepare your meals if you were unable to do it yourself | 1 | 2 | 3 | 4 | 5 | 7 | 9 |

**SECTION D: RELATIONSHIP WITH FATHER OF BABY (FOB) AND HIS MOTHER**

Now, I'd like to ask you some questions about your relationship with the father of your baby (FOB).

D1.  **(HAND R PURPLE CARD SIDE 2)** How often do you have contact with (FOB)? Is it nearly every day, at least once a week, a few times a month, a few times a year, less than once a year or never?

1. NEARLY EVERY DAY (4 OR MORE TIMES A WEEK)

2. AT LEAST ONCE A WEEK (1-3 TIMES)

3. A FEW TIMES A MONTH (1-3 TIMES)

4. A FEW TIMES A YEAR

6. NEVER

5. LESS THAN ONCE A YEAR

7. SKIP

9. DK

**GO TO D3 BELOW**

D2. **(PURPLE CARD SIDE 2)** How often would you like to have contact with (FOB), is it nearly every day, at least once a week, a few times a month, a few times a year, less than once a year or never?

1. NEARLY EVERYDAY (4 OR MORE TIMES A WEEK)

2. AT LEAST ONCE A WEEK (1-3 TIMES)

3. A FEW TIMES A MONTH (1-3 TIMES)

4. A FEW TIMES A YEAR

6. NEVER

5. LESS THAN ONCE A YEAR

7. SKIP

9. DK

D3. **(HAND R GREEN CARD SIDE 1)** How would you describe your relationship with (FOB) before you became pregnant? Was it very close, somewhat close, sometimes close and sometimes cold, somewhat cold, or very cold?

1. VERY CLOSE

5. VERY COLD

2. SOMEWHAT CLOSE

3. SOMETIMES CLOSE/ SOMETIMES COLD

4. SOMEWHAT COLD

7. SKIP

9. DK

D3a. **(GREEN CARD SIDE 1)** How would you describe your current relationship with (FOB)? Is it…

1. VERY CLOSE

5. VERY COLD

2. SOMEWHAT CLOSE

3. SOMETIMES CLOSE/ SOMETIMES COLD

4. SOMEWHAT COLD

7. SKIP

9. DK

D4. Has your relationship with (FOB) changed since the pregnancy?

1. YES

5. NO

**GO TO D6**

D5. In what way has the relationship changed?

1. _________________________________________________________________
2. _________________________________________________________________
3. _________________________________________________________________

Please look at the **YELLOW CARD** **SIDE 2** for the next question. On a scale of 1-7, with 1 being completely satisfied and 7 being completely dissatisfied, tell me how you feel.

D6. How satisfied are you with your current relationship with (FOB)?

DK

SKIP

**1 2 3 4 5 6 7**

COMPLETELY NEITHER COMPLETELY

SATISFIED SATISFIED NOR DISSATISFIED

DISSATISFIED

D9. **(HAND R GREEN CARD SIDE 2)** Please indicate the extent to which you agree or disagree with the following statements.

| FOB Relationship Scale | STRONGLY AGREE  (1) | SOMEWHAT  AGREE  (2) | NEITHER  AGREE NOR DISAGREE  (3) | SOMEWHAT  DISAGREE  (4) | STRONGLY DISAGREE  (5) | SKIP  (7) | DON’T KNOW  (9) |
| --- | --- | --- | --- | --- | --- | --- | --- |
| D9a. (FOB) is always there when I need him. |  |  |  |  |  |  |  |
| D9b. I feel that I can tell (FOB) just about everything. |  |  |  |  |  |  |  |
| D9c. I feel that (FOB) and I can share our problems with each other. |  |  |  |  |  |  |  |
| D9d. I feel that (FOB) and I can share our feelings with each other. |  |  |  |  |  |  |  |
| D9e. (FOB) and I are much closer than most couples. |  |  |  |  |  |  |  |
| D9f. I have a lot of respect for (FOB). |  |  |  |  |  |  |  |
| D9g. (FOB) and I have a good relationship |  |  |  |  |  |  |  |
| D9h. (FOB) is someone I can count on for financial support if I need it. |  |  |  |  |  |  |  |
| D9i. (FOB) is someone I can count on to take care of my baby. |  |  |  |  |  |  |  |
| D9j. (FOB) is often critical (disapproving) of me. |  |  |  |  |  |  |  |
| D9k. I sometimes fight or argue with (FOB). |  |  |  |  |  |  |  |
| D9l. My relationship with (FOB) sometimes makes me feel tense. |  |  |  |  |  |  |  |
| D9m. (FOB) often criticizes my friends. |  |  |  |  |  |  |  |
| D9n. (FOB) often criticizes my (mother). |  |  |  |  |  |  |  |

D10. **(HAND R WHITE CARD SIDE 1)** During your relationship, would you say you have provided a lot more support, advice, and help to (FOB) than he has provided to you, you have provided somewhat more than he has provided, it has been about equal, or he has provided somewhat more or a lot more support to you?

1. R. PROVIDED A LOT MORE

2. R PROVIDED SOMEWHAT MORE

3. ABOUT EQUAL

4. FOB PROVIDED SOMEWHAT MORE

5. FOB PROVIDED A LOT MORE

7. SKIP

9. DK

#### RELATIONSHIP WITH FOB’S MOTHER

Next, I'd like to ask you some questions about your relationship with (woman who raised FOB).

**INTERVIEWER CHECKPOINT: Refer to Household Listing**

1. (WOMAN WHO RAISED FOB) LIVES IN HOUSEHOLD **GO TO D12**

5. (WOMAN WHO RAISED FOB) NOT IN HOUSEHOLD

D11. Do you know (woman who raised FOB)?

1. YES

5. NO

2. YES, DECEASED

**GO TO SECTION E**

D11a. **(HAND R PURPLE CARD SIDE 2)** How often do you have contact with her?

1. NEARLY EVERYDAY (4 OR MORE TIMES A WEEK

6. NEVER

5. LESS THAN ONCE A YEAR

2. AT LEAST ONCE A WEEK

3. A FEW TIMES A MONTH

4. A FEW TIMES A YEAR

7. SKIP

9. DK

**GO TO SECTION E**

D12. **(HAND R GREEN CARD SIDE 1)** How would you describe your relationship with (woman who raised FOB) before you became pregnant?

1. VERY CLOSE

5. VERY

COLD

6. DID NOT KNOW HER AT THE TIME

2. SOMEWHAT CLOSE

3. SOMETIMES CLOSE/ SOMETIMES COLD

4. SOMEWHAT COLD

7. SKIP

9. DK

D13. **(GREEN CARD SIDE 1)** How would you describe your current relationship with (woman who raised FOB)?

1. VERY CLOSE

5. VERY

COLD

6. DID NOT KNOW HER AT THE TIME

2. SOMEWHAT CLOSE

3. SOMETIMES CLOSE/ SOMETIMES COLD

4. SOMEWHAT COLD

7. SKIP

9. DK

D14. Has your relationship with (woman who raised FOB) changed since the pregnancy?

1. YES

5. NO

D15. How satisfied are you with your current relationship with (FOB’s mother)? **(YELLOW CARD SIDE 2)**

DK

SKIP

**1 2 3 4 5 6 7**

COMPLETELY NEITHER COMPLETELY

SATISFIED SATISFIED NOR DISSATISFIED

DISSATISFIED

**SECTION E: RELATIONSHIP WITH BABY**

Now I have a few questions about your baby (with FOB).

E3. How satisfied are you with (FOB’s) involvement with the pregnancy? **(YELLOW CARD SIDE 2)**

DK

SKIP

**1 2 3 4 5 6 7**

COMPLETELY NEITHER COMPLETELY

SATISFIED SATISFIED NOR DISSATISFIED

DISSATISFIED

E4. How has (FOB) been involved with the pregnancy? That is, what types of things has he done?

a. _______________________________________________________________________

b. ________________________________________________________________________

c. ________________________________________________________________________

E5. Would you have liked (FOB) to be more involved or less involved with the pregnancy or stay the same?

1. MORE

2. LESS

3. SAME

7. SKIP

9. DK

**GO TO E2 BELOW (PAGE 17)**

E5b. What things do you see keeping (FOB) from being more involved with the pregnancy?

(READ EACH STATEMENT AND CHECK ALL THAT APPLY)

|  | YES  (1) | NO  (5) | SKIP  (7) | DK  (9) |
| --- | --- | --- | --- | --- |
| 1. Money problems |  |  |  |  |
| 2. Your boyfriend/spouse |  |  |  |  |
| 3. Conflict with your boyfriend or spouse |  |  |  |  |
| 4. (FOB) has another girlfriend/spouse |  |  |  |  |
| 5. Conflict with (FOB's) girlfriend or spouse |  |  |  |  |
| 6. (FOB) has too many work responsibilities |  |  |  |  |
| 7. Involvement with his friends |  |  |  |  |
| 8. Responsibilities to other children |  |  |  |  |
| 9. (FOB) feels he is not needed |  |  |  |  |
| 10. Relationship with your mother |  |  |  |  |
| 11. Relationship with you (for example: you are not willing to let FOB see the baby) |  |  |  |  |
| 12. (FOB’S) Personal problems (for example: substance abuse) |  |  |  |  |
| 13. (FOB’S) Health problems |  |  |  |  |
| 14. (FOB) is in prison or jail |  |  |  |  |
| 15. (FOB) lives far away (for example: 25 or more miles) |  |  |  |  |
| 16. Conflict with your father |  |  |  |  |
| 17. Other, please specify___________________________ |  |  |  |  |

E2. How involved do you think (FOB) will be in raising your (baby with FOB)?

1. VERY INVOLVED

2. SOMEWHAT INVOLVED

3. NOT TOO INVOLVED

4. NOT INVOLVED AT ALL

7. SKIP

9. DK

E7. How satisfied are you with the amount of financial help (FOB) gave your baby during the pregnancy? **(YELLOW CARD SIDE 2)**

DK

SKIP

**1 2 3 4 5 6 7**

COMPLETELY NEITHER COMPLETELY

SATISFIED SATISFIED NOR DISSATISFIED

DISSATISFIED

E8. How satisfied are you with the amount of financial help you gave your baby during the pregnancy? **(YELLOW CARD SIDE 2)**

DK

SKIP

**1 2 3 4 5 6 7**

COMPLETELY NEITHER COMPLETELY

SATISFIED SATISFIED NOR DISSATISFIED

DISSATISFIED

**SECTION F: RELATIONSHIP WITH R'S MOTHER**

The next few questions are about your relationship with the woman who raised you.

F1. Did you have contact with your biological mother while you were growing up?

1. YES

5. NO

**GO TO F1b**

F1a. Is your biological mother the woman who raised you?

1. YES

5. NO

**GO TO F2a**

F1b. Is there another woman who was or is like a mother to you?

1. YES

5. NO

**GO TO SECTION G**

F2. What relationship is she to you?

F2a. Is she still living?

1. YES

5. NO

**GO TO F3**

F2b. When did she die?

F3. How many grades of school did (the woman who raised you) finish?

GRADE OF SCHOOL: 00 01 02 03 04 05 06 07

08 09 10 11 12

DK

SKIP

COLLEGE: 13 14 15 16 17+

F3b. Did (the woman who raised you) get her GED or pass a high school equivalency test?

9. DK

7. SKIP

2. NO

1. YES

F4. **(HAND R WHITE CARD SIDE 1)** During your life, would you say you have provided a lot more support, advice, and help to (the woman who raised R), somewhat more, has it been about equal, or has she provided somewhat more or a lot more support to you?

1. R PROVIDED A LOT MORE

2. R PROVIDED SOMEWHAT MORE

3. ABOUT

EQUAL

4. MOTHER PROVIDED SOMEWHAT MORE

5. MOTHER PROVIDED A LOT MORE

7. SKIP

9. DK

F5. **(HAND R GREEN CARD SIDE 1)** How would you describe your relationship with (the woman who raised R) before the pregnancy? Was it...

1. VERY CLOSE

5. VERY

COLD

2. SOMEWHAT CLOSE

3. SOMETIMES CLOSE/ SOMETIMES COLD

4. SOMEWHAT COLD

7. SKIP

9. DK

**INTERVIEWER CHECKPOINT:**

**IF R ANSWERED NO TO F2a (MOTHER/WOMAN WHO IS LIKE A MOTHER**

**TO YOU IS DECEASED) → GO TO SECTION G**

**GO TO SECTION G**

F6. **(GREEN CARD SIDE 1)** How would you describe your current relationship with (the woman who raised R)? Is it …

1. VERY CLOSE

5. VERY

COLD

2. SOMEWHAT CLOSE

3. SOMETIMES CLOSE/ SOMETIMES COLD

4. SOMEWHAT COLD

7. SKIP

9. DK

F7. Does (the woman who raised R) know about the pregnancy?

1. YES

5. NO

**GO TO F8**

F7a. Has your relationship with (the woman who raised R) changed since the pregnancy?

1. YES

5. NO

Please tell me on a scale of 1-7, with one being completely satisfied and seven being completely dissatisfied, how you feel.

F8. **(YELLOW CARD SIDE 2)** How satisfied are you with your current relationship with (the woman who raised R)?

DK

SKIP

**1 2 3 4 5 6 7**

COMPLETELY NEITHER COMPLETELY

SATISFIED SATISFIED NOR DISSATISFIED

DISSATISFIED

F9. **(YELLOW CARD SIDE 2)** How satisfied are you with the amount of help you receive from (the woman who raised R)?

DK

SKIP

**1 2 3 4 5 6 7**

COMPLETELY NEITHER COMPLETELY

SATISFIED SATISFIED NOR DISSATISFIED

DISSATISFIED

**SECTION G: RELATIONSHIP WITH R'S FATHER**

The next few questions are about your relationship with the man who raised you.

G1. Did you have contact with your biological father while you were growing up?

1. YES

5. NO

**GO TO G1b**

G1a. Is your biological father the man who raised you?

1. YES

5. NO

**GO TO G2a**

G1b. Is there another man who was or is like a father to you?

1. YES

5. NO

**GO TO SECTION H**

G2. What relationship is he to you?

G2a. Is he still living?

1. YES

5. NO

**GO TO G3**

G2b. When did he die?

G3. How many grades of school did (the man who raised you) finish?

GRADE OF SCHOOL: 00 01 02 03 04 05 06 07

08 09 10 11 12

COLLEGE: 13 14 15 16 17+

G3b. Did (the man who raised you) get his GED or pass a high school equivalency test?

9. DK

7. SKIP

2. NO

1. YES

**INTERVIEWER CHECKPOINT:**

| **R answered NO to G1b (THERE IS NO MAN WHO IS LIKE A FATHER) → GO TO SECTION H**    **R answered YES to G1b (THERE IS A MAN WHO IS LIKE A FATHER) → GO TO G4** |
| --- |

G4. **(WHITE CARD SIDE 1)** During your life, would you say you have provided more support, advice, and help to (the man who raised R), has it been about equal, or has he provided more support to you?

1. R. PROVIDED A LOT MORE

2. R PROVIDED SOMEWHAT MORE

3. ABOUT

EQUAL

4. FATHER PROVIDED

SOMEWHAT MORE

5. FATHER PROVIDED A LOT MORE

9. DK

7. SKIP

G5. **(HAND R GREEN CARD SIDE 1)** How would you describe your relationship with (the man who raised R) before you became pregnant? Was it very close, somewhat close, sometimes close and sometimes cold, somewhat cold, or very cold?

1. VERY CLOSE

5. VERY COLD

2. SOMEWHAT CLOSE

3. SOMETIMES CLOSE/ SOMETIMES COLD

4. SOMEWHAT COLD

9. DK

7. SKIP

**INTERVIEWER CHECKPOINT:**

**R ANSWERED NO TO G2a (IF FATHER/MAN WHO IS LIKE A FATHER TO YOU IS**

**DECEASED) → GO TO SECTION H**

**OTHER (CONTINUE)**

G6. **(GREEN CARD SIDE 1)** How would you describe your current relationship with (the man who raised R)? Is it...

1. VERY CLOSE

5. VERY COLD

2. SOMEWHAT CLOSE

3. SOMETIMES CLOSE/ SOMETIMES COLD

4. SOMEWHAT COLD

7. SKIP

9. DK

G7. Has your relationship with (the man who raised R) changed since the pregnancy?

1. YES

5. NO

G8. **(YELLOW CARD SIDE 2)** How satisfied are you with your current relationship with (the man who raised R)?

DK

SKIP

**1 2 3 4 5 6 7**

COMPLETELY NEITHER COMPLETELY

SATISFIED SATISFIED NOR DISSATISFIED

DISSATISFIED

###### SECTION H: PERSONAL CHARACTERISTICS

H1. **(HAND R ORANGE CARD SIDE 1)** From time to time, everyone feels sad or blue. I'm going to read a list of statements that express these feelings. I'd like to know how often you have felt this way in the past week.

### CES - D

| During the past week.... | RARELY OR NONE OF THE TIME (LESS THAN 1 DAY)  (0) | SOME OR A LITTLE OF THE TIME (1-2 DAYS)  (1) | OCCASIONALLY OR A MODERATE AMOUNT OF THE TIME (3-4 DAYS)  (2) | MOST OR ALL OF THE TIME  (5-7 DAYS)  (3) | SKIP  (7) | DON’T KNOW  (9) |
| --- | --- | --- | --- | --- | --- | --- |
| H1a. I was bothered by things that usually don't bother me |  |  |  |  |  |  |
| H1b. I did not feel like eating: my appetite was poor |  |  |  |  |  |  |
| H1c. I felt that I could not shake off the blues even with help from my family or friends |  |  |  |  |  |  |
| H1d. I felt that I was just as good as other people |  |  |  |  |  |  |
| H1e. I had trouble keeping my mind on what I was doing |  |  |  |  |  |  |
| H1f. I felt depressed |  |  |  |  |  |  |
| H1g. I felt that everything I did was an effort |  |  |  |  |  |  |
| H1h. I thought my life had been a failure |  |  |  |  |  |  |
| H1i. I felt hopeful about the future |  |  |  |  |  |  |
| H1j. I felt fearful |  |  |  |  |  |  |
| H1k. My sleep was restless |  |  |  |  |  |  |
| H1l. I was happy |  |  |  |  |  |  |
| H1m. I talked less than usual |  |  |  |  |  |  |
| H1n. I felt lonely |  |  |  |  |  |  |
| H1o. People were unfriendly |  |  |  |  |  |  |
| H1p. I enjoyed life |  |  |  |  |  |  |
| H1q. I had crying spells |  |  |  |  |  |  |
| H1r. I felt sad |  |  |  |  |  |  |
| H1s. I felt that people disliked me |  |  |  |  |  |  |
| H1t. I could not get "going" |  |  |  |  |  |  |

H2. Was this week any different from other weeks?

**GO TO H13**

1. YES

5. NO

H3. In what way has it been different?

a.

b.

c.

H13. **(HAND R PINK CARD SIDE 1)** In the next series of questions I will ask you about your feelings and thoughts during the last month. For each question, please indicate how often you felt or thought that way.

Cohen’s Perceived Stress

| In the last month, how often have you... | Never  (1) | Almost Never  (2) | Sometimes  (3) | Fairly Often  (4) | Very Often (5) | Skip (7) | Don’t Know (9) |
| --- | --- | --- | --- | --- | --- | --- | --- |
| 1. ...been upset because of something that happened that you didn’t expect? |  |  |  |  |  |  |  |
| 1. ...felt that you were in control of your life? |  |  |  |  |  |  |  |
| 1. ...felt nervous and stressed out? |  |  |  |  |  |  |  |
| 1. ...dealt successfully with daily hassles? |  |  |  |  |  |  |  |
| 1. ...felt that you were able to successfully handle the important changes occurring in your life? |  |  |  |  |  |  |  |
| 1. ...felt able to handle your personal problems? |  |  |  |  |  |  |  |
| 1. ...felt that things were going your way? |  |  |  |  |  |  |  |
| 1. ...found that you could not deal with all the things that you had to do? |  |  |  |  |  |  |  |
| 1. ...been able to control hassles in your life? |  |  |  |  |  |  |  |
| 1. ...felt that you were on top of things? |  |  |  |  |  |  |  |
| 1. ...gotten angry because of things that happened that were outside of your control? |  |  |  |  |  |  |  |
| 1. ...found yourself thinking about things you need to do? |  |  |  |  |  |  |  |
| 1. ...been able to control the way you spend your time? |  |  |  |  |  |  |  |
| 1. ...felt that you had so many problems that you could not deal with them? |  |  |  |  |  |  |  |

H14. **(HAND R PINK CARD SIDE 2)** Think about the most stressful situation you have had in the past week. By "stressful" I mean something that was troubling to you either because you felt stressed about what happened, or because you had to make a big effort to deal with it. The situation could have involved your family, friends, school, job, or something else important to you.

Think about the details of this stressful situation. That is, where it happened, who was involved,

how you acted, and why it was important to you. You may still be involved in the situation or it could be over. It should just be the most stressful situation that you experienced last week. As you answer each of the following statements, keep this stressful situation in mind. Now, please tell me to what extent you did what the statement describes by saying it does not apply or it was not used, it was used somewhat, it was used quite a bit, or it was used a great deal.

Ways of Coping – Lazarus & Folkman

| In the past week... | DOES NOT APPLY OR IT WAS NOT USED  (1) | USED  SOMEWHAT  (2) | USED QUITE A BIT  (3) | USED A GREAT DEAL  (4) | SKIP  (7) | DON’T KNOW  (9) |
| --- | --- | --- | --- | --- | --- | --- |
| H14a. I did something that I didn't think would work, but at least I was doing something. |  |  |  |  |  |  |
| H14b. I tried to get the person responsible to change his or her mind. |  |  |  |  |  |  |
| H14c. I criticized or lectured myself. |  |  |  |  |  |  |
| H14d. I went along with fate; sometimes I just have bad luck. |  |  |  |  |  |  |
| H14e. I went on as if nothing had happened. |  |  |  |  |  |  |
| H14f. I looked for the silver lining, so to speak; I tried to look on the bright side of things. |  |  |  |  |  |  |
| H14g. I expressed anger to the person(s) who caused the problem. |  |  |  |  |  |  |
| H14h. I tried to forget the whole thing. |  |  |  |  |  |  |
| H14i. I apologized or did something to make up. |  |  |  |  |  |  |
| H14j. I let my feelings out somehow. |  |  |  |  |  |  |
| H14k. I realized that I had brought the problem on myself. |  |  |  |  |  |  |
| H14l. I took a big chance or did something very risky to solve the problem. |  |  |  |  |  |  |
| H14m. I didn't let it get to me; I refused to think too much about it. |  |  |  |  |  |  |
| H14n. I made light of the situation; I refused to get too serious about it. |  |  |  |  |  |  |
| H14o. I stood my ground and fought for what I wanted. |  |  |  |  |  |  |
| H14p. I promised myself that things would be different next time. |  |  |  |  |  |  |

**SECTION I: HEALTH**

Next, we have some questions concerning your health.

I1. How would you rate your physical health at this time? Would you say it is excellent, very good, good, fair, or poor?

1. EXCELLENT

2. VERY GOOD

3. GOOD

4. FAIR

5. POOR

9. DK

7. SKIP

I2. Would you say that your physical health is a lot better, a little better, average, a little worse or a lot worse than most people your age?

1. A LOT BETTER

2. A LITTLE BETTER

3. AVERAGE

4. A LITTLE WORSE

5. A LOT WORSE

9. DK

7. SKIP

I3. Did you have any serious medical problems before your 10th birthday? Please tell me about them.

1. YES

5. NO

7. SKIPPED

8. MISSING/REFUSED

9. DON’T KNOW

Please specify: _________________________________________________________

I4. Other than at birth, were you ever hospitalized before your 10th birthday? Please tell me about that.

1. YES

5. NO

7. SKIPPED

8. MISSING/REFUSED

9. DON’T KNOW

Please specify: _________________________________________________________

I5. Did you have any serious medical problems between your 10th and 18th birthday? Please tell me about them.

1. YES

5. NO

7. SKIPPED

8. MISSING/REFUSED

9. DON’T KNOW

Please specify: _________________________________________________________

I6. Were you ever hospitalized between your 10th and 18th birthday? Please tell me about that.

1. YES

5. NO

7. SKIPPED

8. MISSING/REFUSED

9. DON’T KNOW

Please specify: _________________________________________________________

I7. How would you rate your mental health at this time? Would you say it is excellent, very good, good, fair, or poor?

1. EXCELLENT

2. VERY GOOD

3. GOOD

4. FAIR

5. POOR

7. SKIP

9. DK

I8. Would you say that your mental health is a lot better, a little better, average, a little worse or a lot worse than most people your age?

1. A LOT BETTER

2. A LITTLE BETTER

3. AVERAGE

4. A LITTLE WORSE

5. A LOT WORSE

9. DK

7. SKIP

I9. **(HAND R YELLOW CARD SIDE 2)** Overall, how satisfied are you with your mental health?

DK

SKIP

**1 2 3 4 5 6 7**

COMPLETELY NEITHER COMPLETELY

SATISFIED SATISFIED NOR DISSATISFIED

DISSATISFIED

H4. **(HAND R ORANGE CARD SIDE 2)** The following questions ask about how you have been feeling during the past 30 days.

K-6 Kessler

| During the past 30 days, about how often did you feel… | ALL OF THE TIME  (1) | MOST OF THE TIME  (2) | SOME OF THE TIME  (3) | A LITTLE OF THE TIME  (4) | NONE OF THE TIME  (5) | SKIP  (7) | DON’T KNOW  (9) |
| --- | --- | --- | --- | --- | --- | --- | --- |
| a. …nervous? |  |  |  |  |  |  |  |
| b. …hopeless? |  |  |  |  |  |  |  |
| c. …restless or fidgety? |  |  |  |  |  |  |  |
| d. …so depressed that nothing could cheer you up? |  |  |  |  |  |  |  |
| e. …that everything was an effort? |  |  |  |  |  |  |  |
| f. …worthless? |  |  |  |  |  |  |  |

H5. The last set of questions asked about feelings that might have occurred during the past 30 days. Taking them altogether, did these feelings occur more often in the past 30 days than usual for you, about the same, or less often than usual?

1. MORE OFTEN THAN USUAL…………………………………..**GO TO H5b**
2. ABOUT THE SAME AS USUAL………………………………...**GO TO H6**
3. LESS OFTEN THAN USUAL
4. (IF VOL) NEVER HAVE THESE FEELINGS…………………..**GO TO H6**

H5a. Is that **a lot** less than usual, **somewhat** less, or **only a little less** than usual?

1. A LOT………………………………………………………………..**GO TO H6**
2. SOMEWHAT………………………………………………………..**GO TO H6**
3. A LITTLE…………………………………………………………….**GO TO H6**

H5b. Is that **a lot** more than usual, **somewhat** more, or **only a little more** than usual?

1. A LOT
2. SOMEWHAT
3. A LITTLE

**H6. INTERVIEWER CHECKPOINT**

1. R ANSWERED “NONE OF THE TIME” TO ALL QUESTIONS IN THE H4 SERIES**..GO TO I10**

2. ALL OTHERS CONTINUE

H7. How many days out of the past 30 were you **totally** unable to work or carry out your normal activities because of these feelings?

__________________ NUMBER OF DAYS

**H8. INTERVIEW CHECKPOINT**

1. R ANSWERED “30” IN RESPONSE TO H7………………………….GO TO H10

2. ALL OTHERS

H9. How many days in the past 30 were you able to do only half or less of what you would normally have been able to do because of these feelings?

_________________NUMBER OF DAYS

H10. During the past 30 days, how many times did you see a doctor or other health professional about these feelings?

_______________NUMBER OF TIMES

H11. **(HAND R ORANGE CARD SIDE 2)** During the past 30 days, how often have physical health problems been the main cause of these feelings – **all** of the time, **most** of the time, **some** of the time, **a little** of the time, or **none** of the time?

1. ALL
2. MOST
3. SOME
4. A LITTLE
5. NONE

Now we are going to ask you about some other health issues.

I10. Have you been to a dentist in the past year?

1. YES

5. NO

I11. Do you have a medical doctor who you saw regularly prior to your pregnancy?

1. YES

5. NO

I12. In the past year, have you been to any of these places for health care?

| a) A hospital clinic? | **Yes**  1 | **No**  5 |
| --- | --- | --- |
| b) The local health department? | 1 | 5 |
| c) Planned Parenthood or a family planning clinic? | 1 | 5 |
| d) A private doctor’s office? | 1 | 5 |
| e) An emergency room? | 1 | 5 |
| f) A community mental health center? | 1 | 5 |
| g) Another kind of health care clinic or office?  (Specify:____________________________________________________) | 1 | 5 |

I13a. Do you have a health insurance?

1. YES

5. NO

**GO TO I14**

I13b. What type of health insurance do you have?

___________________________________________ (Specify: private, with job; private, personal pay, Medicare; Medicaid; state program etc.)

I14. During your pregnancy, how often did you drink a beer, glass of wine, or liquor? (READ LIST)

1. NEVER

2. ONCE

3. 2-3 TIMES

4. ONCE A WEEK

5. SEVERAL TIMES A WEEK

6. EVERYDAY

**GO TO I16**

I15. During your pregnancy, when you drank beer, wine, or liquor, about how much did you usually drink on one occasion? (READ LIST)

1. LESS THAN A FULL CAN OR GLASS

2. ONE CAN OR GLASS

3. TWO CANS OR GLASSES

4. THREE CANS OR GLASSES

5. FOUR OR MORE CANS OR GLASSES

I16a. Have you ever smoked cigarettes?

1. YES

5. NO

**GO TO I20**

I16b. Did you smoke cigarettes in the past year?

1. YES

5. NO

**GO TO I20**

I17. How many cigarettes have you smoked during the past week? (READ LIST)

1. NONE

2. LESS THAN DAILY

3. 1-5 CIGARETTES PER DAY

4. ABOUT ½ PACK PER DAY

5. ABOUT ONE PACK PER DAY

6. MORE THAN ONE PACK PER DAY

I18. How many cigarettes did you smoke in the first half of your pregnancy? (READ LIST)

1. NONE

2. LESS THAN DAILY

3. 1-5 CIGARETTES PER DAY

4. ABOUT ½ PACK PER DAY

5. ABOUT ONE PACK PER DAY

6. MORE THAN ONE PACK PER DAY

I19. How old were you when you started smoking regularly?

_____ _____ years old

# I would like to find out about your use of street drugs. Before we begin, I want to remind you that this information is entirely confidential and will not be shared with your doctors or nurses, or with anyone else. Your name will not be placed on this questionnaire.

|  | **PART I** | | **PART II** |
| --- | --- | --- | --- |
| I20. Have you ever used … | Ever used | | When was the last time you used? |
|  | ***IF NO,***  ***SKIP PART II*** |  | ____ ___/ ___ ___/ __ ___  Month Day Year |
|  | NO | YES |  |
| a. Marijuana, hashish, pot, grass? | 5 | 1 |  |
| b. Cocaine, coke, crack? | 5 | 1 |  |
| c. Heroin? | 5 | 1 |  |
| d. Methadone? | 5 | 1 |  |
| e. Any other drugs such as amphetamines or LSD?  SPECIFY______________ | 5 | 1 |  |

Now I would like to ask some questions specifically about your pregnancy.

I22b. It’s hard for many women to remember when their pregnancy began, but let’s try to do so.

When was your last menstrual period?

____ ____/ ____ ____/ ___ ____ (Last Menstrual Period)

Month DAY YEAR

**If R knows the month but not the day, probe and enter:**

First week of month (44), middle of month (55), end of month (66)?

(**IF PRENATAL INTERVIEW AND R CANNOT REMEMBER, ASK):**

I22c. How many weeks pregnant are you?

__________ (Weeks pregnant)

I would like to know more about your weight during your childhood. Please tell me these answers to the best of your recollection.

| I23. How much did you weigh at birth? | I23a______ _____ pounds  77 = skipped  88 = not asked  99 = don’t know  I23b______ _____ ounces  44 = quarter pound  55 = half a pound  66 = three-quarters of a pound  77 = skipped  88 = not asked  99 = don’t know | I23a. __ __  I23b. __ __ |
| --- | --- | --- |
| I24a. At age 10, would you say you were…? ***READ RESPONSES***  **(HAND R GREY CARD SIDE 1)** | Very underweight………………………………1  Skinny…………………………………………...2  Just right………………………..……………….3  A little heavy but not overweight……………...4  Overweight………………………………….…..5  Extremely overweight……………………..……6  Skipped 7  NA (__ missing / __ refused) 8  Don’t Know 9 | I24a. ___ |
|  | |  |
|  | |  |
| I25a. At age 18, would you say you were…? ***READ RESPONSES***  **(HAND R GREY CARD SIDE 1)** | Very underweight…………………………….….1  Skinny…………………………………………….2  Just right………………………..………………...3  A little heavy but not overweight……………….4  Overweight……………………………………..5  Extremely overweight………………………….6  Skipped 7  NA (__ missing / __ refused) 8  Don’t Know 9 | I25a. ___ |
| I25b. Please look at the picture. Of the body types shown, which one best represents your body type at age 18? **(HAND R FLORESCENT GREEN CARD SIDE 1)** | | I25b. ___ |

| I25c. How much did you weigh at age 18? | Pounds………………….. ____ ____ ____  Skipped 777  NA (__ missing / __ refused) 888  Don’t Know 999 |
| --- | --- |
| I26. How much did you weigh before you became pregnant? | Pounds………………….. ____ ____ ____  Skipped 777  NA (__ missing / __ refused) 888  Don’t Know 999 |
| I27. How much weight did you gain during this pregnancy? | Pounds ___ ___ ___  Skipped 777  NA (__ missing / __ refused) 888  DK 999 |

Think back to the five years before this pregnancy.

I28a. How much did you weigh at your heaviest when you were not pregnant? ____ ____ ____ pounds

I28b. How much did you weigh at your lightest when you were not pregnant? ____ ____ ____ pounds

Now I have some questions about your activities during this pregnancy.

I30. How many times per day during this pregnancy did you usually climb stairs?

__________ __________ (# of Times Per Day)

I31. How many minutes on average, have you exercised per day during this pregnancy?

_________ _________ ________ (# of Minutes Per Day) ***IF ANSWER IS 0 MINUTES,***

***GO TO I32***

I31a. What activities have you done for exercise during this pregnancy? (***LIST FIRST THREE)***

1. WALKING

2. JOGGING/ RUNNING

3. AEROBICS/ AEROBIC DANCING

4. SOCIAL/ NIGHTCLUB DANCING

5. BICYCLE RIDING

6. SWIMMING/

WATER EXERCISES

7. BASKETBALL/ VOLLEYBALL

8. OTHER (please specify):

___________________________

I32. During your pregnancy, how many minutes per day did you spend walking outside of the home that was not part of an exercise program? For example, walking for “a purpose” such as to get to the bus, to the store, etc.

__________ __________ (# of Minutes Per Day)

Now I’m going to ask you some questions that are a little more personal. These questions are about douching. When I say “douching”, I mean washing or cleansing inside your vagina.

| I33. At any time before or during your pregnancy, did you ever douche? | Yes 1  No (***SKIP TO I36***) 5  Skipped 7  NA(__ missing / __ refused) 8  Don’t Know 9 | I33. ___ |
| --- | --- | --- |
|  |  |  |
| I34. During the 6 months before you knew you were pregnant, did you ever douche? | Yes 1  No (***SKIP TO I35***) 5  Skipped 7  NA(__ missing / __refused) 8  Don’t Know 9 | I34. ___ |
| I34a. How often? ***READ RESPONSES*** | Less Than Once a Month 1  Once or Twice a Month 2  Three or Four Times a Month 3  More Than Four Times a Month 4  Skipped 7  NA(__ missing / __ refused) 8  Don’t Know 9 | I34a.___ |
| I35. During your pregnancy, did you ever douche? | Yes 1  No (***SKIP TO I36***) 5  Skipped 7  NA(__ missing / __ refused) 8  Don’t Know 9 | I35. ___ |
| I35a. How often? ***READ RESPONSES*** | Less Than Once a Month 1  Once or Twice a Month 2  Three or Four Times a Month 3  More Than Four Times a Month 4  Skipped 7  NA(__ missing / __ refused) 8  Don’t Know 9 | I35a.___ |

| I36. During your pregnancy did you experience any of the following: | No | Yes | SK | NA | DK |  |
| --- | --- | --- | --- | --- | --- | --- |
| a. unusual vaginal odor | 5 | 1 | 7 | 8 | 9 | I36a.___ |
| b. unusual vaginal itching or burning | 5 | 1 | 7 | 8 | 9 | I36b.___ |
| 1. unusual vaginal discharge   (Probe – color, consistency):  __________________________________ | 5 | 1 | 7 | 8 | 9 | I36c.___ |
| d. doctor’s diagnosis of vaginal infection | 5 | 1 | 7 | 8 | 9 | I36d.___ |
| 1. bleeding or spotting   (Probe – color, consistency):  __________________________________ | 5 | 1 | 7 | 8 | 9 | I36e.___ |
| ***IF YES, ASK***:  e1. any bleeding or spotting 1^st^ trimester? | 5 | 1 | 7 | 8 | 9 | I36e1.__ |
| e2. any bleeding or spotting 2^nd^ trimester? | 5 | 1 | 7 | 8 | 9 | I36e2.__ |
| e3. any bleeding or spotting 3^rd^ trimester? | 5 | 1 | 7 | 8 | 9 | I36e3.__ |

| I37. Have you ever had an abnormal pap smear? | Yes 1  No (***SKIP TO I42***) 5  Skipped 7  NA (__ missing / __ refused) 8  Don’t Know 9 | I37. ___ |
| --- | --- | --- |

I38. What was the official diagnosis? ____________________________________________________

I39. What year was it? ___________________________

I40. How was it treated? ______________________________________________________________

I41. Who treated it (Gynecologist, Family doctor…)? ________________________________________

Now I’m going to ask some questions about your experiences at the prenatal clinic or doctor’s office for prenatal visits.

I42. How often did (FOB) go with you to the prenatal clinic or on prenatal office visits with the doctor? Would you say… ***READ RESPONSES***

1. **All** of the time

2. **Most** of the time

3. **Some** of the time

4.  **None** of the time

|  | **PART I** | | **PART II** | |
| --- | --- | --- | --- | --- |
| I43. Since you have been pregnant, has anyone at the prenatal clinic talked to you about any of the following topics: | Talked | | Did They Tell You What to Do About It? | |
|  | ***IF NO,***  ***SKIP PART II*** |  |  | |
|  | NO | YES | NO | YES |
| a. what you should eat | 5 | 1 | 5 | 1 |
| b. how much you should sleep | 5 | 1 | 5 | 1 |
| c. any chronic health problems  you may have | 5 | 1 | 5 | 1 |
| d. contractions/labor pains when  they are too early | 5 | 1 | 5 | 1 |
| e. your baby’s movement, if it  slows down | 5 | 1 | 5 | 1 |
| f. what you should do about  smoking | 5 | 1 | 5 | 1 |
| g. what you should do about  alcohol use | 5 | 1 | 5 | 1 |
| h. what you should do about  using prescription drugs | 5 | 1 | 5 | 1 |

*PART II – PROBE (i.e. what kinds of foods, amount – hours of sleep)

I44. Before you became pregnant did you **ever** have any of the following chronic health problems?

NO YES

Asthma 5 1

Diabetes 5 1

Hypertension 5 1

Thyroid problems 5 1 Specify: Hypo Hyper

Other health problems 5 1 Specify: ________________

that required treatment _______________________

_______________________

_______________________

**SECTION J: RELIGION**

J1. **(HAND R PURPLE CARD SIDE 2)** How often do you attend religious services? Would you say:

1. Nearly everyday 2. At least once a week 3. A few times a month

4. A few times a year 5. Less than once a year 6. Never **(GO TO J4)**

7. Skip 8. NA: Volunteers not a church member. 9. DK

J2. **(HAND R YELLOW CARD SIDE 2)** On a scale of 1-7, how satisfied are you with the quality of the relationships you have with the people in your church or place of worship?

DK

SKIP

**1 2 3 4 5 6 7**

COMPLETELY NEITHER COMPLETELY

SATISFIED SATISFIED NOR DISSATISFIED

DISSATISFIED

J4. How religious would you say you are? Would you say...

1. VERY RELIGIOUS

2. SOMEWHAT RELIGIOUS

3. NOT TOO RELIGIOUS

4. NOT AT ALL RELIGIOUS

**GO TO**

**SECTION K**

J5b. What is your current religious denomination? ___________________________________

For the next few questions, please tell me how often you do each. **(HAND R FLORESCENT GREEN CARD SIDE 2)**

|  | VERY OFTEN (1) | FAIRLY OFTEN (2) | NOT TOO OFTEN (3) | NEVER (4) |
| --- | --- | --- | --- | --- |
| J7. How often do you read religious books or other religious materials? Would you say... |  |  |  |  |
| J8. How often do you watch or listen to religious programs on TV or radio? |  |  |  |  |
| J9. How often do you listen to religious music? |  |  |  |  |
| J10. How often do you pray? |  |  |  |  |
| J11. How often do you ask someone to pray for you? |  |  |  |  |

**SECTION K: RACIAL IDENTITY, SOCIALIZATION AND DISCRIMINATION**

K1. **(HAND R BLUE CARD SIDE 1)** Now I would like to know how you feel about being Black. Please tell me if you strongly agree, somewhat agree, somewhat disagree, or strongly disagree with each of the following statements.

Multidimensional Inventory of Black Identity (Sellers 1997)

|  | Strongly Agree  (1) | Agree  (2) | Disagree  (3) | Strongly Disagree  (4) | Skip  (7) | Don’t Know  (9) |
| --- | --- | --- | --- | --- | --- | --- |
| a) In general, being Black is an important part of my self-image. |  |  |  |  |  |  |
| b) My destiny is tied to the destiny of other Black people. |  |  |  |  |  |  |
| c) I have a strong attachment to other Black people. |  |  |  |  |  |  |
| d) Being Black is an important part of who I am. |  |  |  |  |  |  |
| e) I feel good about Black people. |  |  |  |  |  |  |
| f) I am happy that I am Black. |  |  |  |  |  |  |
| g) I am proud to be Black. |  |  |  |  |  |  |
| h) I feel that the Black community has made valuable contributions to this society. |  |  |  |  |  |  |
| i) In general, society respects Black people. |  |  |  |  |  |  |
| j) In general, other racial groups view Blacks in a positive way. |  |  |  |  |  |  |
| k) Society views Black people as an asset (something good). |  |  |  |  |  |  |
| l) In general, other racial groups respect Black people. |  |  |  |  |  |  |
| m) I often regret that I am Black. |  |  |  |  |  |  |
| n) Blacks are often considered good by others. |  |  |  |  |  |  |
| o) In general, other racial groups view Blacks as competent (capable) people. |  |  |  |  |  |  |

K2. **(HAND R BLUE CARD SIDE 2)** These next questions ask you to think about experiences that some people have as they go about their daily lives. Please **first** determine how often you have each experience because of your race or racism. Next, tell me how much it bothers you when the experience happens. I will ask you to tell me about what you experienced in the year before your pregnancy as well as what you’ve experienced during your pregnancy.

***Daily Life Experiences (Race & Bother Scales; DLE-B)***

| **In the previous year** | **How often because of race did you experience…**  ***READ RESPONSES*** | How much did it  bother you?  ***READ RESPONSES*** |  |
| --- | --- | --- | --- |
|  |
| K2. Being ignored, overlooked, or not given service (in a restaurant, store, etc.) | K2a. ____  Never 1  Less than Once a yr …..2  A Few Times a Year 3  About Once a Month 4  A Few Times a Month 5  Once a Week or More 6  Skipped 7  NA 8  Don’t Know 9 | K2b. ____  Never happened to me 1  Doesn’t bother me at all 2  Bothers me a little 3  Bothers me somewhat 4  Bothers me a lot 5  Bothers me extremely 6  Skipped 7  NA 8  Don’t Know 9 |  |
| K3. Being treated rudely or disrespectfully |  |  |  |
| K4. Being accused of something or treated suspiciously |  |  |  |
| K5. Others reacting to you as if they were afraid or intimidated |  |  |  |
| K6. Being observed or followed while in public places |  |  |  |
| K7. Being treated as though you were “stupid” |  |  |  |
| K8. Your ideas or opinions being minimized, ignored, or devalued. |  |  |  |
| K9. Overhearing or being told an offensive joke or comment |  |  |  |
| K10. Being insulted, called a name, or harassed |  |  |  |
| K11. Others expecting your work to be inferior |  |  |  |
| K12. Not being taken seriously |  |  |  |
| K13. Being left out of a conversation or activities |  |  |  |
|  | Never 1  Less than Once a yr …..2  A Few Times a Year 3  About Once a Month 4  A Few Times a Month 5  Once a Week or More 6  Skipped 7  NA 8  Don’t Know 9 | Never happened to me 1  Doesn’t bother me at all 2  Bothers me a little 3  Bothers me somewhat 4  Bothers me a lot 5  Bothers me extremely 6  Skipped 7  NA 8  Don’t Know 9 |  |
| K14. Being treated in an “overly” friendly superficial way |  |  |  |
| K15. Being avoided, others moving away from you physically |  |  |  |
| K16. Being mistaken for someone who serves others (i.e. secretary, janitor, maid) |  |  |  |
| K17. Being stared at by strangers |  |  |  |
| K18. Being laughed at, made fun of, or taunted |  |  |  |
| K19. Being mistaken for someone else of your same race (who may not look like you at all) |  |  |  |
| K20. Being asked to speak for or represent your entire racial/ethnic group (e.g. “What to __ people think?”) |  |  |  |
| K21. Being considered fascinating or exotic by others |  |  |  |
|  |  |  |  |
|  |  |  |  |

**K22. RESPONSES TO RACE-RELATED EXPERIENCES (RRE)**. **(HAND R LIGHT YELLOW CARD SIDE 1)**

Now, think about when you have experiences in different areas of your life (such as work or school), or as you go about your daily life, that you believe happen because of your race.

| K22a. How much does it bother you when something happens to you because of your race? *READ RESPONSES ALOUD* | Bothers You Extremely 1  Bothers You A Lot 2  Bothers You Somewhat 3  Bothers You A Little 4  Doesn’t Bother You At All 5  Skipped 7  NA (__ missing / __ refused) 8  Don’t Know 9 | K22a. ___ |
| --- | --- | --- |

| **(HAND R ORANGE CARD SIDE 2)** | | |
| --- | --- | --- |
| K22h. How often do you confront the person(s) involved when something happens because of your race? *READ RESPONSES ALOUD* 8. _____ | Always 1  Most of the Time 2  Sometimes 3  A Little Bit of the Time 4  Very Rarely or Never 5  Skipped 7  NA (__ missing / __ refused) 8  Don’t Know 9 | K22h. ___ |
| K22i. How often do you take some other kind of action when something happens because of your race? *READ RESPONSES ALOUD* | Always 1  Most of the Time 2  Sometimes 3  A Little Bit of the Time 4  Very Rarely or Never 5  Skipped 7  NA (__ missing / __ refused) 8  Don’t Know 9 | K22i. ___ |
| K22j. How often do you say or do nothing at all when something happens because of your race? *READ RESPONSES ALOUD* | Always 1  Most of the Time 2  Sometimes 3  A Little Bit of the Time 4  Very Rarely or Never 5  Skipped 7  NA (__ missing / __ refused) 8  Don’t Know 9 | K22j. ___ |
| K22k. How often do you talk to someone about it when something happens because of your race? *READ RESPONSES ALOUD* | Always 1  Most of the Time 2  Sometimes 3  A Little Bit of the Time 4  Very Rarely or Never 5  Skipped 7  NA (__ missing / __ refused) 8  Don’t Know 9 | K22k. ___ |
| K22l. How often do you doubt that what happened was really because of your race? *READ RESPONSES ALOUD* | Always 1  Most of the Time 2  Sometimes 3  A Little Bit of the Time 4  Very Rarely or Never 5  Skipped 7  NA (__ missing / __ refused) 8  Don’t Know 9 | K22l. ___ |

***Major Experiences of Discrimination (From NSAL) – YES Health and*** DAS '95

| K23. **(HAND R LIGHT YELLOW CARD SIDE 2)** In the following questions, we are interested in the way other people have treated you or your *beliefs* about how other people have treated you. Can you tell me if *any* of the following has ever happened to you: | |
| --- | --- |
|  | K23(2). What do you think was the main reason for this experience?  1 – Your Ancestry or National Origins  2 – Your Gender  3 – Your Race  4 – Your Age  5 – Your Height or Weight  6 – Your shade of skin color  11- Other (SPECIFY) ______________________ |
| a1. At any point in your  life, have you ever been  unfairly fired?  1 – yes →  5 – no  7 - NA | a2.  1 2 3 4 5 6 11 ______________________ |
| b1. For unfair reasons,  have you ever not been  hired for a job?  1 – yes →  5 – no  7 - NA | b2.  1 2 3 4 5 6 11 ______________________ |
| c1. Have you ever been  unfairly denied a  promotion?  1 – yes →  5 – no  7 - NA | c2.  1 2 3 4 5 6 11 ______________________ |
| d1. Have you ever been  unfairly stopped,  searched, questioned,  physically threatened  or abused by the police?  1 – yes →  5 – no  7 – NA | d2.  1 2 3 4 5 6 11 ______________________ |
| e1. Have you ever been  unfairly discouraged by  a teacher or advisor  from continuing your  education?  1 – yes →  5 – no  7 – NA | e2.  1 2 3 4 5 6 11 ______________________ |
| f1. Have you ever been  unfairly prevented from  moving into a  neighborhood because  the landlord or a realtor  refused to sell or rent  you a house or apartment?  1 – yes →  5 – no  7 - NA | f2.  1 2 3 4 5 6 11 ______________________ |
| g1. Have you ever moved  into a neighborhood where  neighbors made life  difficult for you or your  family?  1 – yes →  5 – no  7 – NA | g2.  1 2 3 4 5 6 11 ______________________ |
| h1. Have you ever been  unfairly denied a bank  loan?  1 – yes →  5 – no  7 – NA | h2.  1 2 3 4 5 6 11 ______________________ |
| i1. Have you ever received  service from someone  such as a plumber or  car mechanic that was  worse than what other  people get?  1 – yes →  5 – no  7 - NA | i2.  1 2 3 4 5 6 11 ______________________ |

K24. How many of your friends are Black — all, most, some, a few, or none?

1. ALL

2. MOST

3. SOME

4. A FEW

5. NONE

***Shade of Skin Color - DAS ’95***

K25. **(HAND R LIGHT PURPLE CARD SIDE 1)** Compared to most Black people, what shade of skin color do you have? Would you say very dark brown, dark brown, medium brown, light brown or very light brown?

1 – Very dark brown

2 – Dark brown

3 – Medium brown

4 – Light brown

5 – Very light brown

**INTERVIEWER CHECKPOINT** (See **A2 and A2d**)

1. IF A2 IS EQUAL TO ‘1’ or’2’ (MARRIED OR LIVING WITH PARTNER) → **GO TO K26**

2. IF A2d IS EQUAL TO 1 (GOING WITH SOMEONE) → **GO TO K26**

3. ALL OTHERS GO TO **K27**

K26. **(HAND R LIGHT PURPLE CARD SIDE 1)** Compared to most Black people, what shade of skin color does your spouse/partner have? Would you say very dark brown, dark brown, medium brown, light brown or very light brown?

1 – Very dark brown

2 – Dark brown

3 – Medium brown

4 – Light brown

5 – Very light brown

6 – Spouse/partner not Black

***Shades of Color treatment – Kendrick Brown (NSAL)***

| K27. **(HAND R LIGHT PURPLE CARD SIDE 2)** How often would you say... | Very Often  (1) | Fairly Often  (2) | Not Too Often  (3) | Hardly Ever  (4) | Never (5) |
| --- | --- | --- | --- | --- | --- |
| 1. … that Whites treat you badly because of the shade of your skin color? Would you say very often, fairly often, not too often, hardly ever, or never? |  |  |  |  |  |
| 1. ... that Blacks treat you badly because of the shade of your skin color? |  |  |  |  |  |
| 1. ...(men/women SAY OPPOSITE SEX OF R) find you attractive because of your skin color? |  |  |  |  |  |

**SECTION L: SERVICE UTILIZATION**

# L1. What is the most serious problem that you have had since knowing about the pregnancy?

_______________________________________________________________________

_______________________________________________________________________

L2. Over the past year, have there been any services, such as WIC, job training, health or mental services that you have needed but have not gotten?

1. YES

5. NO

**GO TO L2b**

L2a. In order of importance, which services have you needed but have not gotten?

- - - 1. ________________________________________________________________
      2. ________________________________________________________________
      3. ________________________________________________________________

L2b. Have you received any of the following services since you have been pregnant?

NO YES

Alcohol counseling 5 1

Mental health services 5 1

Housing assistance 5 1

Major health services 5 1

Social work services 5 1

**SECTION M: SCHOOLING**

Now, we'd like to ask you some questions about your education.

M3. Have you graduated from high school or passed a high school equivalency test?

1. HIGH SCHOOL DIPLOMA

2. HIGH SCHOOL EQUIVALENCY

5. NO

**GO TO M3b**

M3a. In what year did you (graduate from high school or get your high school equivalency)?

_________________________

M3b. Have you had any other schooling?

1. YES

5. NO

**GO TO M1a**

M3c. What kind?

M1. Are you going to any high school, college, vocational school, job training program, or any other

school at this time (even though school may be out for the summer)?

1. YES

5. NO

**GO TO M2**

M1a. Are you planning to go back to school soon?

1. YES

5. NO

M2. How many grades of school have you finished?

GRADE OF SCHOOL: 00 01 02 03 04 05 06 07

08 09 10 11 12

DK

SKIP

COLLEGE: 13 14 15 16 17+

M2b. Did you get your GED or pass a high school equivalency test?

9. DK

7. SKIP

2. NO

1. YES

M4. To get a picture of different families’ financial situation, we would like to know the general range of income of all families we interview. Thinking about your family’s total income from all sources, about how much was your family’s income last year? **(GIVE R LIGHT GREEN CARD SIDE 1)**

1. <$5,000

2. $5,000 – 9,999

3. 10,000 – 14,999

4. 15,000 – 19,999

5. 20,000 – 24,999

6. 25,000 - 29,999

7. 30,000 – 34,999

8. 35,000 – 39,999

9. 40,000 – 44,999

10. 45,000 – 49,999

11. 50,000 – 54,999

12. 55,000 – 59,999

13. 60,000 – 69,999

14. 70,000 – 79,999

15. 80,000 – 89,999

16. 90,000 – 99,999

17. $100,000 or more

DK

SKIP

M6. Please tell me your current age?

__________________

**SECTION N: RESIDENTIAL ENVIRONMENT/NEIGHBORHOOD QUESTIONS**

Finally, we have some questions about what it’s like to live in your neighborhood. By neighborhood we mean the area around where you live and around your house. It may include places you shop, religious or public institutions, or a local business district. It is the general area around your house where you might perform routine tasks, such as shopping, going to the park, or visiting with neighbors.

Now I’m going to read some statements about things that people in your neighborhood may or may not do.

# NEIGHBORHOOD SOCIAL COHESION & TRUST (a-g), HEALTHY FOOD AVAILABILITY (h-i), AND WALKING ENVIRONMENT (j-o)

N1. **(HAND R LIGHT GREEN CARD SIDE 2)** For each of these statements, please tell me whether you strongly agree, agree, disagree, or strongly disagree.

|  | Strongly agree | Agree | Neither agree nor disagree | Disagree | Strongly disagree | Skip | DK |
| --- | --- | --- | --- | --- | --- | --- | --- |
| a. I live in a close-knit neighborhood | 1 | 2 | 3 | 4 | 5 | 7 | 9 |
| b. People in my neighborhood are willing to help their neighbors | 1 | 2 | 3 | 4 | 5 | 7 | 9 |
| c. People in my neighborhood generally don’t get along with each other | 1 | 2 | 3 | 4 | 5 | 7 | 9 |
| d. People in my neighborhood do not share the same values | 1 | 2 | 3 | 4 | 5 | 7 | 9 |
| e. People in my neighborhood can be trusted | 1 | 2 | 3 | 4 | 5 | 7 | 9 |
| f. People in this neighborhood are willing to help women if they are in trouble | 1 | 2 | 3 | 4 | 5 | 7 | 9 |
| g. People in this neighborhood are willing to help pregnant women | 1 | 2 | 3 | 4 | 5 | 7 | 9 |
| h. A large selection of fresh fruits and vegetables is available in my neighborhood | 1 | 2 | 3 | 4 | 5 | 7 | 9 |
| i. A large selection of low fat products is available in my neighborhood | 1 | 2 | 3 | 4 | 5 | 7 | 9 |
| j. It is pleasant to walk in my neighborhood | 1 | 2 | 3 | 4 | 5 | 7 | 9 |
| k. The trees in my neighborhood provide enough shade | 1 | 2 | 3 | 4 | 5 | 7 | 9 |
| l. In my neighborhood it is easy to walk to places | 1 | 2 | 3 | 4 | 5 | 7 | 9 |
| m. I often see other people walking in my neighborhood | 1 | 2 | 3 | 4 | 5 | 7 | 9 |
| n. I often see other people exercise in my neighborhood | 1 | 2 | 3 | 4 | 5 | 7 | 9 |
| o. There are stores within walking distance of my home | 1 | 2 | 3 | 4 | 5 | 7 | 9 |

**NEIGHBORHOOD SOCIAL DISORDER**

N2. **(HAND R LIGHT ORANGE CARD SIDE 1)** I’m going to read a list of things that are problems in some neighborhoods. For each, please tell me how much of a problem it is in your neighborhood.

|  | A big problem | Somewhat of a problem | Not a problem | Skip | DK |
| --- | --- | --- | --- | --- | --- |
| a. How much of a problem is litter, broken glass, or trash on the sidewalks and streets? Would you say it is a big problem, somewhat of a problem, or not a problem in your neighborhood | 1 | 2 | 3 | 7 | 9 |
| b. How much of a problem is graffiti on buildings and walls? | 1 | 2 | 3 | 7 | 9 |
| c. How much of a problem are vacant or deserted houses or storefronts? | 1 | 2 | 3 | 7 | 9 |
| d. How much of a problem is drinking in public? | 1 | 2 | 3 | 7 | 9 |
| e. How much of a problem is people selling or using drugs? | 1 | 2 | 3 | 7 | 9 |
| f. How much of a problem is groups of teenagers or adults hanging out in the neighborhood and causing trouble? | 1 | 2 | 3 | 7 | 9 |
| g. How much of a problem is noise in the neighborhood? | 1 | 2 | 3 | 7 | 9 |
| h. How much of a problem is yelling and fighting? | 1 | 2 | 3 | 7 | 9 |

**NEIGHBORHOOD PERSONAL VICTIMIZATION**

| N3a. While you have lived in this neighborhood, has anyone ever used violence, such as in a mugging, fight, or sexual assault, against you or any member of your household, anywhere in your neighborhood? | Yes 1  No 5  Skipped 7  NA (__ missing / __ refused) 8  Don’t Know 9 | a. ___ |
| --- | --- | --- |

**PERCEIVED NEIGHBORHOOD DANGER AND SAFETY**

N4. **(HAND R LIGHT GREEN CARD SIDE 2)** I am going to read you some statements people sometimes make. For each, please tell me whether you strongly agree, agree, disagree, or strongly disagree with each.

|  | Strongly agree | Agree | Neither agree nor disagree | Disagree | Strongly disagree | Skip | DK |
| --- | --- | --- | --- | --- | --- | --- | --- |
| a. Many people in your neigh-borhood are afraid to go out at night. (would you say you strongly agree, agree, disagree, or strongly disagree?) | 1 | 2 | 3 | 4 | 5 | 7 | 9 |
| b. There are areas of this neighborhood where everyone knows “trouble” is expected. | 1 | 2 | 3 | 4 | 5 | 7 | 9 |
| c. You’re taking a big chance if you walk in this neighborhood alone after dark. | 1 | 2 | 3 | 4 | 5 | 7 | 9 |
| d. I feel safe walking in my neigh-borhood | 1 | 2 | 3 | 4 | 5 | 7 | 9 |
| e. Violence is a problem in my neighborhood | 1 | 2 | 3 | 4 | 5 | 7 | 9 |
| f. I feel very safe from crime in my neighborhood | 1 | 2 | 3 | 4 | 5 | 7 | 9 |

**NEIGHBORHOOD-BASED SOCIAL TIES**

N5. **(HAND R LIGHT ORANGE CARD SIDE 2)** I’m going to ask you a few questions about who you know in your neighborhood. Not counting those who live with you…

|  | None | One or Two | Three to Five | Six to Ten | Ten or More | Skip | DK |
| --- | --- | --- | --- | --- | --- | --- | --- |
| a. How many of your relatives or in-laws or kin live in your neighborhood? Would you say none, one or two, three to five, six to ten, or ten or more? | 1 | 2 | 3 | 4 | 5 | 7 | 9 |
| b. How many friends do you have in your neighborhood? | 1 | 2 | 3 | 4 | 5 | 7 | 9 |
| c. How many friends do you have who live outside of your neighborhood? | 1 | 2 | 3 | 4 | 5 | 7 | 9 |

**NEIGHBORHOOD OVERALL QUALITY**

|  | Excellent | Good | Fair | Poor | Skip | DK |
| --- | --- | --- | --- | --- | --- | --- |
| N6a. Overall, how would you rank your neighborhood as a place to live? | 1 | 2 | 3 | 4 | 7 | 9 |

**LENGTH OF TIME IN NEIGHBORHOOD**

N7a. How many times have you moved in the last year? __________

| N7b. How long, in years and months, have you lived in this neighborhood where you currently live? | # of years ___ ___  # of months ___ ___  Skipped 77  NA (__ missing / __ refused) 88  Don’t Know 99 | b. ____ |
| --- | --- | --- |

| N7c. How would you describe the place where you live now? ***IF NECESSARY, USE ANSWER CATEGORIES TO PROBE*** | Apartment in a House or  Garden Apartment (1-4 stories) 01  High Rise Apartment (more than 4 floors) 02  Rowhouse or Duplex 03  Detached Single Family House 04  Shelter (***SKIP TO N8***) 05  Other (***SPECIFY***) 07  Specify: __________________________________ | c.___ |
| --- | --- | --- |
| N7d. Do you or someone in your household own your place with a mortgage, own without a mortgage, or is it rented? | Own with a mortgage 1  Own without a mortgage (***SKIP TO N8***) 2  Rented (***SKIP TO N8***) 3  Other (***SPECIFY***) 4  Specify: ___________________________________ | d.___ |
| N7i. Was your house foreclosed on during your pregnancy? i.___  1. YES  5. NO  5. NO  1. YES  **SKIP TO N8**  N7j. Was your house foreclosed on at any point in the past 2 years? j.___ | | |

N7e. Were you afraid of losing your house to foreclosure during your pregnancy? e. ___

Would you say...

1. VERY AFRAID

2. AFRAID

3. NOT VERY AFRAID

4. NOT AFRAID AT ALL

N7f. Have you ever missed a mortgage payment on your current home? f.___

1. YES

5. NO

**SKIP TO N8**

N7g.When did you last miss a mortgage payment on this home? (Month/Year) ___ ___/ ___ ____

N7h. How many payments have you ever missed on the mortgage for your current home? ____ ____

**RESIDENTIAL HISTORY**

We are interested in where you live, and where you have lived in the past, to understand how those influences in the past might have influenced your health. ***PROBE FURTHER IF P.O. BOX IS GIVEN OR IF DETAILED ADDRESS IS NOT GIVEN.***

N8. What is your current address?

| Apartment or House Number: __________________________________________________________  Street: ____________________________________________________________________________  City: ______________________________________ State: ___________ Zip: _____________  Phone number #1: ___________________________________________________________________  Phone number #2: ___________________________________________________________________  Phone number #3: ___________________________________________________________________  Email: _____________________________________________________________________________  (Do not ask. See N9f below.) Did the respondent experience a foreclosure at this address? (Circle one)    yes no skipped don’t know |
| --- |

N9. To better study this issue, we’d also like to have the addresses for all the places you have lived in the past 2 years. If you cannot remember or do not know, do you know the cross streets close to where you lived or any landmarks (churches, schools) that may still exist, near where you lived or do you know the city and state, or zip code?

| a. □ Same as current address  Apartment or House Number: __________________________________________________________  Street/Cross street/Landmarks:__________________________________________________________  City: ______________________________________ State: ___________ Zip: _____________  (Do not ask. See N9f below.) Did the respondent experience a foreclosure at this address? (Circle one)    yes no skipped don’t know |
| --- |
| b. □ Same as current address  Apartment or House Number: __________________________________________________________  Street/Cross street/Landmarks:__________________________________________________________  City: ______________________________________ State: ___________ Zip: _____________  (Do not ask. See N9f below.) Did the respondent experience a foreclosure at this address? (Circle one)    yes no skipped don’t know |
| c. □ Same as current address  Apartment or House Number: __________________________________________________________  Street/Cross street/Landmarks:__________________________________________________________  City: ______________________________________ State: ___________ Zip: _____________  (Do not ask. See N9f below.) Did the respondent experience a foreclosure at this address? (Circle one)    yes no skipped don’t know |
| d. □ Same as current address  Apartment or House Number: __________________________________________________________  Street/Cross street/Landmarks:__________________________________________________________  City: ______________________________________ State: ___________ Zip: _____________  (Do not ask. See N9f below.) Did the respondent experience a foreclosure at this address? (Circle one)    yes no skipped don’t know |
| e. □ Same as current address  Apartment or House Number: __________________________________________________________  Street/Cross street/Landmarks:__________________________________________________________  City: ______________________________________ State: ___________ Zip: _____________  (Do not ask. See N9f below.) Did the respondent experience a foreclosure at this address? (Circle one)    yes no skipped don’t know |

**Interviewers: Ask the next question if the respondent is currently experiencing or have experienced foreclosure during pregnancy at the addresses she provided above. If the answer is YES, go back to the where the address is listed above and indicate Yes or No at the end of each section.**

N9f. Did you experience a foreclosure while you were staying at any of the addresses you provided? (Circle one)

yes no skipped don’t know

N10. What was your address 10 years ago?

| □ Same as current address  Apartment or House Number: __________________________________________________________  Street: ____________________________________________________________________________  City: ______________________________________ State: ___________ Zip: _____________ |
| --- |

N10a. If you cannot remember or do not know, do you know the cross streets close to where you lived?

N10b. If you still do not know, can you recall any landmarks (churches, schools) that may still exist, near where you lived?

N10c. If you still do not know, do you know the city and state, or zip code?

N11. What was your address when you were aged 18?

| □ Same as current address  Apartment or House Number: __________________________________________________________  Street: ____________________________________________________________________________  City: ______________________________________ State: ___________ Zip: _____________ |
| --- |

N11a. If you cannot remember or do not know, do you know the cross streets close to where you lived?

N11b. If you still do not know, can you recall any landmarks (churches, schools) that may still exist, near where you lived?

N11c. If you still do not know, do you know the city and state, or zip code?

N12. What was your address when you were aged 10?

| □ Same as current address  Apartment or House Number: __________________________________________________________  Street: ____________________________________________________________________________  City: ______________________________________ State: ___________ Zip: _____________ |
| --- |

N12a. If you cannot remember or do not know, do you know the cross streets close to where you lived?

N12b. If you still do not know, can you recall any landmarks (churches, schools) that may still exist, near where you lived?

N12c. If you still do not know, do you know the city and state, or zip code?

N13. What was your address when you were born?

| □ Same as current address  Apartment or House Number: __________________________________________________________  Street: ____________________________________________________________________________  City: ______________________________________ State: ___________ Zip: _____________ |
| --- |

N13a. If you cannot remember or do not know, do you know the cross streets close to where you lived?

N13b. If you still do not know, can you recall any landmarks (churches, schools) that may still exist, near where you lived?

N13c. If you still do not know, do you know the city and state, or zip code?

**PAST NEIGHBORHOOD CONTEXT**

Now I’d like you to think about the neighborhood you lived in when you were around the **age of 10**.

**PAST NEIGHBORHOOD SOCIAL CONTROL**

N14. **(HAND R LIGHT BLUE CARD SIDE 1)** For each of the following, please tell me if it is very likely, likely, unlikely, or very unlikely that people from your neighborhood **when you were 10 years old** would act in the following manner.

|  | Very likely | Likely | Neither likely nor unlikely | Unlikely | Very unlikely | Skip | Don’t Know |
| --- | --- | --- | --- | --- | --- | --- | --- |
| a. If a group of neighborhood children were skipping school and hanging out on a street corner, how likely is it that your neighbors would have done something about it?  Would you say it was …  [READ LIST] | 1 | 2 | 3 | 4 | 5 | 7 | 9 |
| b. If some children were spray-painting graffiti on a local building, how likely is it that your neighbors would have done something about it? | 1 | 2 | 3 | 4 | 5 | 7 | 9 |
| c. If a child was showing disrespect to an adult, how likely is it that people in your neighborhood would have scolded that child? | 1 | 2 | 3 | 4 | 5 | 7 | 9 |
| d. If there was a fight in front of your house and someone was being beaten or threatened, how likely is it that your neighbors would break it up? | 1 | 2 | 3 | 4 | 5 | 7 | 9 |

**PAST NEIGHBORHOOD SOCIAL DISORDER**

N15. **(HAND R LIGHT ORANGE CARD SIDE 1)** I’m going to read a list of things that are problems in some neighborhoods. For each, please tell me how much of a problem it was in the neighborhood you lived in **when you were 10 years old**.

|  | A big problem | Somewhat of a problem | Not a problem | Skip | Don’t Know |
| --- | --- | --- | --- | --- | --- |
| a. How much of a problem was litter, broken glass, or trash on the sidewalks and streets? Would you say it was… [READ LIST] | 1 | 2 | 3 | 7 | 9 |
| b. How much of a problem was graffiti on buildings and walls? | 1 | 2 | 3 | 7 | 9 |
| c. How much of a problem were vacant or deserted houses or storefronts? | 1 | 2 | 3 | 7 | 9 |
| d. How much of a problem was drinking in public? | 1 | 2 | 3 | 7 | 9 |
| e. How much of a problem was people selling or using drugs? | 1 | 2 | 3 | 7 | 9 |
| f. How much of a problem were groups of teenagers or adults hanging out in the neighborhood and causing trouble? | 1 | 2 | 3 | 7 | 9 |

**PAST NEIGHBORHOOD PERSONAL VICTIMIZATION**

| N16. While you lived in the neighborhood when you were **aged 10**, did anyone ever use violence, such as in a mugging, fight, or sexual assault, against you or any member of your household, anywhere in your neighborhood? | Yes 1  No 5  Skipped 7  NA (__ missing / __ refused) 8  Don’t Know 9 | N16. ___ |
| --- | --- | --- |

N18. Those are all of the questions that I have. Now that you know the questions that we wanted to ask, is there anything else important in your life that you think we should know about?

a. _______________________________________________________________________

b. _______________________________________________________________________

c. _______________________________________________________________________

Pt ID_______________

N17. Knowing more about your childhood health and environment may be important in studying this problem. Your mother will be better able to answer these questions than you. We are asking you to provide us with your mother’s most current address and telephone number. We will use this to send a letter to your mother inviting her to participate. We will call her on the telephone, and if she is willing to participate we will then ask her to answer questions about your childhood. We expect the interview to last no more than 15 minutes. There are no risks to your mother from being interviewed; we will send her a $20 Target store gift card to thank her. [EMPHASIZE THAT THE INTERVIEW IS NOT PERSONAL INFORMATION AND THAT IT IS VERY SHORT AND EVEN THOUGH YOU THINK MOTHER MAY SAY NO WE WOULD LIKE TO TRY BECAUSE IT IS IMPORTANT TO THE STUDY]

Mother’s information

Agree to provide………………………………………………...1

Refuse to provide……………………………………………….2

Mother is deceased……………………………………………..3

Mother is institutionalized………………………………………4

Mother is too ill…………………………………………………..5

Mother is unable due to dementia, Alzheimer’s, etc…………6

Skipped…………………………………………………………...7

Not applicable…………………………………………………….8

Don’t know………………………………………………………..9

Mother’s Name: _________________________________________

Apartment or House Number: _____________________________________________________

Street: _______________________________________________________________________

City _________________________________ State: _________ Zip: _______________

Telephone #: _________________________________ Email: ___________________________

Is there a second address or phone number which we should also use?

Apartment or House Number: _____________________________________________________

Street: _______________________________________________________________________

City _________________________________ State: _________ Zip: _______________

Telephone #: _________________________________

**CLOSING STATEMENT**

Thank you for participating in this study and for sharing your experiences with this pregnancy with us.

**EXACT TIME NOW: ________** January 11, 2011

**SECTION O: INTERVIEWER OBSERVATIONS**

O1. Was R suspicious about the study before the interview?

1. YES, VERY SUSPICIOUS

3. YES, SOMEWHAT SUSPICIOUS

5. NO, NOT AT ALL SUSPICIOUS

O2. The respondent's attitude during the interview was:

1. COOPERATIVE, HELPFUL

2. NEUTRAL, RELAXED

3. NERVOUS, UNCERTAIN

4. ANTAGONISTIC

O3. The respondent's attitude at the end of the interview was:

1. NO CHANGE FROM THE BEGINNING OF THE INTERVIEW

2. MORE COOPERATIVE, MORE HELPFUL

3. LESS COOPERATIVE, LESS HELPFUL

O4. Please describe any ambiguous or conflicting situation that you want the study staff or coding to

know about:

NONE

O5. Were there any other serious problems with the interview, such as R's difficulty in hearing or understanding the questions, etc., which affected the quality of the interview?

NONE

O6. How much trouble did the respondent have in expressing himself?

1. A GREAT DEAL OF TROUBLE

2. A LOT OF TROUBLE

3. NOT TOO MUCH TROUBLE

4. NO TROUBLE AT ALL

O7. Was anyone else present during the interview?

1. YES, MOST OF IW

2. YES, HALF OR MORE OF IW

3. YES, BUT INFREQUENT

5. NO

**GO TO O8**

O7a. Who was that? (CHECK ALL THAT APPLY):

1. CHILD(REN) UNDER 10

2. CHILD(REN) 10 OR OVER

3. SPOUSE

4. OTHER FRIENDS OR RELATIVES

O7b. How much did they influence or interfere with the interview?

1. VERY LITTLE

2. SOMEWHAT

3. A GREAT DEAL

O8. Thumbnail Sketch

January 11, 2011
